# Spaceflight Enhances Cell Aggregation and Random Budding in *Candida albicans*

**DOI:** 10.1371/journal.pone.0080677

**Published:** 2013-12-04

**Authors:** Aurélie Crabbé, Sheila M. Nielsen-Preiss, Christine M. Woolley, Jennifer Barrila, Kent Buchanan, James McCracken, Diane O. Inglis, Stephen C. Searles, Mayra A. Nelman-Gonzalez, C. Mark Ott, James W. Wilson, Duane L. Pierson, Heidemarie M. Stefanyshyn-Piper, Linda E. Hyman, Cheryl A. Nickerson

**Affiliations:** 1 School of Life Sciences, Arizona State University, Tempe, Arizona, United States of America; 2 Center for Infectious Diseases and Vaccinology, The Biodesign Institute, Arizona State University, Tempe, Arizona, United States of America; 3 Department of Immunology and Infectious Disease, Montana State University, Bozeman, Montanta, United States of America; 4 Department of Biology, Oklahoma City University, Oklahoma City, Oklahoma, United States of America; 5 Department of Microbiology and Immunology, Program in Molecular Pathogenesis and Immunity, Tulane University Health Sciences Center, New Orleans, Louisiana, United States of America; 6 Diabetes and Obesity Center, University of Louisville, Louisville, Kentucky, United States of America; 7 Department of Genetics, Stanford University Medical School, Stanford, California, United States of America; 8 Wyle Science, Technology and Engineering Group, Houston, Texas, United States of America; 9 Biomedical Research and Environmental Sciences Division, NASA Johnson Space Center, Houston, Texas, United States of America; 10 Department of Biology, Villanova University, Villanova, Pennsylvania, United States of America; 11 Astronaut Office, NASA Johnson Space Center, Houston, Texas, United States of America; 12 Boston University School of Medicine, Boston, Massachusetts, United States of America; Institute of Microbiology, Switzerland

## Abstract

This study presents the first global transcriptional profiling and phenotypic characterization of the major human opportunistic fungal pathogen, *Candida albicans*, grown in spaceflight conditions. Microarray analysis revealed that *C. albicans* subjected to short-term spaceflight culture differentially regulated 452 genes compared to synchronous ground controls, which represented 8.3% of the analyzed ORFs. Spaceflight-cultured *C. albicans*–induced genes involved in cell aggregation (similar to flocculation), which was validated by microscopic and flow cytometry analysis. We also observed enhanced random budding of spaceflight-cultured cells as opposed to bipolar budding patterns for ground samples, in accordance with the gene expression data. Furthermore, genes involved in antifungal agent and stress resistance were differentially regulated in spaceflight, including induction of ABC transporters and members of the major facilitator family, downregulation of ergosterol-encoding genes, and upregulation of genes involved in oxidative stress resistance. Finally, downregulation of genes involved in actin cytoskeleton was observed. Interestingly, the transcriptional regulator Cap1 and over 30% of the Cap1 regulon was differentially expressed in spaceflight-cultured *C. albicans*. A potential role for Cap1 in the spaceflight response of *C. albicans* is suggested, as this regulator is involved in random budding, cell aggregation, and oxidative stress resistance; all related to observed spaceflight-associated changes of *C. albicans*. While culture of *C. albicans* in microgravity potentiates a global change in gene expression that could induce a virulence-related phenotype, no increased virulence in a murine intraperitoneal (i.p.) infection model was observed under the conditions of this study. Collectively, our data represent an important basis for the assessment of the risk that commensal flora could play during human spaceflight missions. Furthermore, since the low fluid-shear environment of microgravity is relevant to physical forces encountered by pathogens during the infection process, insights gained from this study could identify novel infectious disease mechanisms, with downstream benefits for the general public.

## Introduction

The presence of opportunistic pathogens in the normal flora of astronauts, in combination with their compromised immune system during spaceflight missions, puts this population at particular risk for infectious disease [1]–[4]. *Candida* species are commensal organisms that are found on human skin, in the oral cavity, and in the gastrointestinal, urogenital, and vaginal tracts [5] and are consistently isolated from the spaceflight crew and environment [6]–[8]. These microorganisms become pathogenic under specific circumstances, which can lead to various infectious diseases ranging in severity from superficial mucous membrane infections (i.e., thrush) to life-threatening disseminated candidiasis [9]. Immunocompromised patients are at particular risk of developing *Candida* infections [9].

The risk for infectious diseases in astronauts becomes even more significant given previous reports that spaceflight culture conditions globally alter the virulence and/or gene expression of obligate and opportunistic bacterial pathogens [10]–[12]. Two independent spaceflight experiments demonstrated that mice infected with spaceflight-grown *Salmonella enterica* serovar Typhimurium (*S.* Typhimurium) exhibited decreased time to death and LD_50_ values when compared to mice challenged with identical synchronous ground control cultures [11], [12]. Analysis of global transcriptomic and proteomic expression patterns of *S.* Typhimurium grown in spaceflight conditions revealed that 167 transcripts and 73 proteins were altered during culture in the microgravity environment of spaceflight [11], and identified a central regulatory role for the evolutionarily conserved RNA-binding protein Hfq. Hfq is an Sm-like (LSm) RNA chaperone that serves as a master regulator of bacterial responses to environmental stress, primarily by regulating gene expression at the post-transcriptional level through the pairing of mRNA transcripts with cognate small non-coding RNAs [13]–[19]. Spaceflight also alters the *hfq* regulon in *Pseudomonas aeruginosa* [10], and is involved in the spaceflight-analogue response of *S*. Typhimurium, *P. aeruginosa* and *Staphylococcus aureus* [20]–[22]. Spaceflight-analogue conditions are obtained through culturing of microorganisms in rotating bioreactors, termed rotating wall vessels (RWV). In the RWV, cells experience low fluid-shear forces while being in continuous suspension, which mimics aspects of the unique microgravity environment [11], [23]–[25]. This specific growth environment is termed low shear modeled microgravity (LSMMG) [22].

The response of eukaryotic microorganisms to spaceflight and spaceflight-analogue conditions has been previously reported. *Saccharomyces cerevisiae* has been extensively studied since the early years of the space program. The first flight experiment with this organism was conducted in 1962 (reviewed in [26]). Detailed analyses indicated that yeast cells responded to microgravity by undergoing metabolic (e.g. increase in phosphate uptake [27]) and phenotypic changes (e.g. increase in number and distribution of bud scars [28]–[30]). A recent report showed enhanced production of the biochemical molecule S-adenosyl-L-methionine (SAM) in spaceflight-cultured *S. cerevisiae* [31]. Knowledge gained from these studies led to the engineering of a SAM-overproducing strain of *S. cerevisiae*, with potential industrial applications. Moreover, studies describing the response of *S. cerevisiae* to spaceflight-analogue conditions in the RWV showed major phenotypic alterations in response to this environment [32]. Specifically, *S. cerevisiae* grown in LSMMG conditions displayed increased cell clumping (or flocculation) and a random budding phenotype as compared to the bipolar budding pattern of the same cells grown in the control orientation of the RWV bioreactor [32], [33].

While, to our knowledge, no reports exist on the response of *C. albicans* to culture under true spaceflight conditions, studies have documented the response of this organism to ground-based spaceflight-analogue conditions in the RWV [34], [35]. When *C. albicans* was cultured in LSMMG, this organism displayed increased randomness in the budding pattern, which is similar to the phenotype observed for *S. cerevisiae* during culture under the same conditions. In addition, while *C. albicans* existed as a predominantly yeast form when cultured under control conditions in the RWV bioreactor, increased filamentation and biofilm formation were observed when grown under LSMMG as determined by microscopy and morphology-specific gene expression profiling [34], [35]. *C. albicans* can transition from budding yeast to a filamentous (hyphal) form, which is responsive to environmental stressors and contributes to the organism's virulence [36]–[39]. Consistent with the conversion of *C. albicans* cells to a filamentous form, a concomitant increase in expression of filamentous-specific genes that are also suggestive of biofilm formation was observed in response to LSMMG [34], [35], [40], [41].

In addition to the importance of spaceflight research for infectious disease risk assessment during short and long-term missions, studying the behavior of *C. albicans* to spaceflight and spaceflight-analogue culture conditions has important clinical applications [42], [43]. Indeed, the low fluid shear forces to which microorganisms are exposed in spaceflight and spaceflight-analogue cultures are relevant to environmental conditions encountered during their lifecycles on Earth, including in the gastrointestinal, respiratory, and urogenital tracts of the host [42]–[45]. Since we currently lack a complete understanding of the infection process of this medically important pathogen and there is an urgent need for novel therapeutic approaches to control *C. albicans* infections [40], [41], insights gained from microgravity research holds potential to discover new infectious disease mechanisms and benefit the general public on Earth.

The current study describes the response of the most prominent fungal human pathogen, *C. albicans*, to spaceflight culture conditions, flown as part of the NASA Space Shuttle Atlantis Mission STS-115. In this report, we analyzed the global transcriptional profile and performed phenotypic analysis of *C. albicans* during short-term growth in spaceflight conditions. To our knowledge, this is the first report describing the effects of spaceflight culture on the global gene expression and phenotypic changes of a eukaryotic pathogen.

## Experimental Procedures

### Ethics statement

Research was conducted in compliance with applicable animal care guidelines at the NASA Kennedy Space Center (KSC) under approved NASA KSC IACUC Protocol # FLT-06-050.

### Strains, media and growth conditions

*C. albicans* strain SC5314 was used in all experiments. Prior to flight, 6×10^6^ cells grown in YPD medium were suspended in 0.5 mL sterile ddH_2_O and loaded into specialized spaceflight hardware, termed Fluid Processing Apparatuses (FPA) (**Figure S1**), as described previously [10]. Briefly, growth was initiated in flight (nine days post launch) by addition of 2 mL YPD to the fungal suspension (termed *activation*). Cultures were grown in spaceflight conditions or synchronous ground control conditions for 25 hours at ambient temperature (23°C). Subsequently, cells were fixed for RNA, proteins and morphological imaging by addition of 2.5 mL RNA Later II reagent (Ambion, Austin, TX) (termed *termination*). For infection studies, assessment of cell viability and fixation for scanning electron microscopy (SEM), 2.5 mL YPD medium was added instead of RNA Later II fixative. All samples were returned at ambient temperature, and Shuttle landing occurred 12 days post launch. Two and a half hours after landing at Kennedy Space Center, the culture samples fixed in RNA Later II were recovered, removed from the FPA, and stored at −80°C. The viable cell samples were counted by plating on solid medium. A portion of the sample was fixed in 4% glutaraldehyde (16%; Sigma, St. Louis, MO) for SEM analysis, and the remainder of the sample was immediately used for virulence studies in mice. For all studies, flight cultures were compared to synchronous control cultures grown under identical conditions on the ground at Kennedy Space Center using coordinated activation and termination times (via real time communications with the Shuttle crew) in an insulated room that maintained identical temperature and humidity as on the Shuttle (Orbital Environmental Simulator) (synchronous ground controls).

### Virulence

The *C. albicans* dose for infection was obtained by pooling samples from eight FPAs for either flight or ground control samples, respectively, followed by centrifugation (1500 g, 5 min) and resuspension in sterile PBS. Six to eight week old female Balb/c mice (housed in the Animal Facility at the Space Life Sciences Lab at Kennedy Space Center) were injected intraperitoneally (i.p.) (Kretschmar et al., 1999) with a single lethal dose (1×10^8^) of *C. albicans* cells harvested from either spaceflight (within 2.5 hours after Shuttle landing) or synchronous ground cultures that were resuspended in 0.5 mL sterile PBS [11]. Ten mice were used per test condition and infected mice were monitored every 6–12 hours for 14 days.

### Microscopy

All electron microscopy was performed on an XL30 FEI/Philips environmental scanning electron microscope (ESEM). As mentioned above, flight and ground samples were fixed in 4% glutaraldehyde post-landing and stored at 4°C until processing and analysis. Prior to analysis, samples were placed in filtration units containing a polycarbonate membrane with 0.4 µm pore size (Poretics Corporation), gently rinsed three times in filter-sterilized milli-Q water, and then dehydrated with graded alcohol series to 100% ethanol. The polycarbonate filters containing the cells were placed on double-sided carbon tape that was mounted onto stubs and dried overnight in a dry chamber. Next, samples were sputter coated with gold-palladium prior to imaging. Image J (http://rsbweb.nih.gov/ij/) was used to determine the average cell length/width and surface area, based on the analysis of 143 and 197 cells imaged with SEM for ground and spaceflight samples respectively. The individual cell measurements are provided as supplemental data (**Table S1**).

Light microscopic analysis was performed on RNA Later II-fixed samples, using a Zeiss Axiovert microscope (magnification 100, 400× and 630×). Two biological replicates for flight and ground cultures were imaged. To determine average cell cluster size, five random images at magnification 100× were analyzed per biological replicate and per condition. Cells within the ten largest cell clusters were counted per image, and the average over the five microscopic images was determined.

### Flow cytometry

Flow cytometry was performed using a FACS Calibur (Becton Dickinson). *C. albicans* flight and ground cultures (biological duplicate), stored in RNA later II at −80°C were diluted in PBS and subjected to analysis by flow cytometry. A forward scatter threshold was established at 700 to distinguish yeast cells from cell clusters. A population of yeast cells grown in liquid culture at 30°C (no cell clusters) was used to establish this threshold, in which at least 99% of the yeast population fell below the threshold. As forward scatter is proportional to cell size, events with forward scatter greater than the established threshold were considered cell aggregates. For each sample, 10,000 events were acquired at an analysis rate of approximately 500 events per second. All data analysis was performed with Cell Quest software (Becton Dickinson).

### RNA extraction, quantification and microarray analysis

Four independent flight and ground samples were thawed and cells were counted manually using a hemocytometer. Yeast cells were disrupted by homogenization in the presence of glass beads in a Mini-Beadbeater-8™ (Biospec Products) and RNA was isolated using the RNeasy Micro kit (Qiagen). RNA quality and quantity were evaluated using the Nanodrop technology (Thermo Scientific) and an Agilent 2100 bioanalyzer (Agilent Technologies). Samples were processed at the Microarray Core Facility at Washington University (St. Louis, MO) [46], [47]. Briefly, first strand cDNA was generated by oligo-dT primed reverse transcription (Superscript II; Invitrogen), following the manufacturer's instructions. For RNA expression level comparison, samples were paired and concentrated using Microcon YM30 microconcentrators (Millipore) according to the manufacturer's protocol. Next, each sample pair was resuspended in Formamide-based hybridization buffer (vial 7-Genisphere), Array 50 dT blocker (Genisphere), and RNase/DNase-free water. Primary and secondary hybridizations were carried out in a sequential manner following standard protocols [46], [47]. A dye-swap analysis was performed as well, and the data was not significantly different from the data set with the initial dye choice. To prevent fluorophore degradation, the arrays were treated with Dyesaver (Genisphere). Slides were scanned on a Perkin Elmer ScanArray Express HT scanner to detect Cy3 and Cy5 fluorescence. Laser power is kept constant for Cy3/Cy5 scans and PMT is varied for each experiment based on optimal signal intensity with lowest possible background fluorescence. Gridding and analysis of images was performed using ScanArray v3.0 (Perkin Elmer). Background intensity values were imported into Partek Genomic Suite (Partek, Inc.). The median value of each set of replicate spots from each array was used. Data was log2 transformed and quantile normalized [48]. Three-way ANOVA analysis was then performed on the data using treatment (flight vs. ground), dye, and experimental data as factors. Flight to ground linear contrast was performed with ANOVA. False Discovery Rate was controlled using the Step Up method [49]. Analysis was initially restricted to genes that had high intensity on the array and were differentially expressed by at least 2-fold with a confidence interval of 95%. Where indicated, genes with less than a 2.0 fold increase and less than a 95% confidence interval were considered. While the gene expression list was initially based on predicted ORFs annotated in assembly 19 of the *C. albicans* SC5314 genome, it was updated according to the most recent version (assembly 21) at CGD, with regard to gene model merges and gene deletions. The full description of the microarray analysis and the complete microarray data set have been deposited at the Gene Expression Omnibus (GEO) website under accession number GSE50881. The Candida Genome Database (CGD) Gene Ontology (GO) Slim Mapper was used to group differentially expressed genes according to function (biological process). In order to determine statistical significance of enriched categories, the GO Term Finder was used [50]. For the GO Term Finder analysis, the data set was filtered for genes with GO annotations (i.e., 273 out of 452 genes). The GO Term Finder ‘process’ categorization was utilized for these studies unless otherwise noted.

### Quantitative real time PCR (qRT-PCR) analysis

RNA was isolated as described above. One microgram RNA per sample was converted to cDNA using the MonsterscriptTM 1st-strand cDNA synthesis kit (Epicenter), and subsequently diluted ten times in nuclease-free water. Quantitect SYBR Green Master mix (Qiagen) was used to assess differential gene expression with quantitative real time PCR (qRT-PCR), according to the manufacturer's protocol. An overview of primers used in this study is provided in **Table 1**. The qRT-PCR reactions were performed in a RealPlex 2 system (Eppendorf). A melting curve was run at the end of each reaction to test for the presence of a single PCR product. The qRT-PCR reaction product was run on a 3% agarose gel in the presence of a low molecular weight DNA ladder (BioLabs), to assess primer specificity. CT values were exported using the Eppendorf Database tool, where after the delta delta CT method [51] was adopted to determine relative gene expression between different test conditions. The average of four housekeeping genes was used for normalization (*ACT1*, *PMA1*, *RIP*, *RPP2B*) [52]. All chosen housekeeping genes were not differentially expressed based on microarray analysis. Two biological replicates of *C. albicans* grown in spaceflight and ground control conditions were analyzed with qRT-PCR in technical duplicate.

**Table 1:** Primers used for qRT-PCR analysis.

Gene	Category	Forward primer (5′ – 3′)	Reverse primer (5′ – 3′)
*ALS1* *	Biofilm	CAACAGGCACCTCAGCATCTAC	CTCCACCAGTAACAGATCCACTAGTAA
*CAP1*	Transcriptional regulator	ACGTTCACGGTATGCCCTTT	TTCTACACCAAGAATTAAACAACCA
*ERG6*	Antifungal drug resistance	GCTACCGTTCATGCTCCAGT	ACACGAATTGAACACCCCCA
*YTH1*	Filamentation	TAACGGGCATAGCACTCGTC	ACAATTCTTGTCCCCAGGGC
*HSP31*	Stress resistance	TGCAACCACAAGAGGCTTAAC	CAAAACAGCAGGCCAACCAA
*GPX2*	Stress resistance	ACAATCATCAATGGGCAACGAG	AACCCACTTCACCAGGCTTT
*ACT1* *	Normalization	TTTCATCTTCTGTATCAGAGGAACTTATTT	ATGGGATGAATCATCAAACAAGAG
*PMA1* *	Normalization	TTGCTTATGATAATGCTCCATACGA	TACCCCACAATCTTGGCAAGT
*RIP* *	Normalization	TGTCACGGTTCCCATTATGATATTT	TGGAATTTCCAAGTTCAATGGA
*RPP2B* *	Normalization	TGCTTACTTATTGTTAGTTCAAGGTGGTA	CAACACCAACGGATTCCAATAAA

*[52], other primers were designed in this study

## Results

### Gene expression

#### General observations

Whole genome expression profiling was used to identify gene expression alterations in *C. albicans* in response to culture in spaceflight conditions as compared to identical synchronous ground controls. The *C. albicans* microarrays used to assess differential gene expression between flight and ground samples included 6,346 of the 6,742 predicted ORFs annotated in assembly 19 of the *C. albicans* SC5314 genome (**Table S1**) [50]. Of those 6,346 ORFs, there were 5,432 that exhibited a robust response suitable for statistical analysis. Data analysis was restricted to genes that had high intensity on the array and were differentially expressed by at least 2-fold and a p-value <0.05. Of these, 452 (or 8.3% of the analyzed ORFs) were differentially expressed in response to spaceflight culture conditions; 279 were upregulated (61.7%), and 173 were downregulated (38.3%) in the flight samples as compared to ground controls (**Table 2**).

**Table 2:** Differentially regulated genes of *C. albicans* grown in spaceflight conditions as compared to ground control (p<0.05, fold-change >2).

Column ID	Ratio (FLT vs. GRD)	Gene name	Gene function	P-value
UPREGULATED GENES				
orf19.2462_800	12.36	PRN3	RNA pol II transcription cofactor	1.52E-05
orf19.1976_183	11.82	TRX1	thioredoxin II	7.22E-06
orf19.4654_100	9.18		hypothetical protein	3.73E-07
orf19.2428.2*	7.91	POL	RNA-directed DNA polymerase	3.18E-06
orf19.4873_58	7.67		hypothetical protein	6.42E-07
orf19.4653_226	7.51		hypothetical protein	8.89E-08
orf19.4784_2733	6.18	CRD1	copper-transporting P1-type ATPase	5.94E-06
orf19.3643_1045	5.98		hypothetical protein	1.14E-03
orf19.2369.1	5.78	ATX1	antioxidant and copper/iron homeostasis protein	2.21E-04
orf19.633_479	5.73		putative methyltransferase	1.45E-04
orf19.3722_1630	5.72	FAP1	FKBP12-associated protein | transcription factor homolog	1.85E-06
orf19.2989_630	5.52		glycerate/formate- dehydrogenase	1.12E-03
orf19.3114_112	5.37	PUS5	pseudouridylate synthase	7.35E-05
orf19.3902_108	5.37		hypothetical protein	1.93E-02
orf19.3115_540	5.23		hypothetical protein	7.27E-07
orf19.5735.3	5.12		polyprotein of Tca5 retrotransposon	8.32E-06
orf19.4274_526	5.00	PUT1	proline oxidase	1.47E-05
orf19.207_3938	4.95		extremely serine rich protein	8.36E-05
orf19.3721_54	4.87		hypothetical protein	3.34E-04
orf19.1277_1084	4.76		hypothetical protein	1.49E-04
orf19.2157_168	4.76	NAG2	N-acetylglucosamine-6-phosphate deacetylase	8.79E-05
orf19.3120_767	4.72		highly conserved hypothetical protein, possible ABC transporter	3.15E-02
orf19.3668_781	4.56	HGT2	hexose transporter	9.20E-05
orf19.7283_265	4.48		hypothetical protein	1.17E-04
orf19.265_519	4.46		hypothetical protein	1.33E-03
orf19.4779_1348	4.34		multidrug-resistance transporter	1.61E-05
orf19.716_12	4.22		similar to pore-forming bacterial Septicolysin	4.75E-05
orf19.7042_467	4.11		hypothetical protein	2.13E-03
orf19.7098_396	4.07		transcription factor	8.01E-04
orf19.4526_520	3.96	HSP30	plasma membrane heat shock protein	4.22E-05
orf19.4045_129	3.93	EST1	EST1-like bcy1 Suppressor	3.18E-04
orf19.5180_89	3.89	PRX1	regulation of redox homeostasis	6.51E-03
orf19.101_672	3.84	RIM9	low similarity to a regulator of sporulation	5.22E-04
orf19.7300_80	3.71		hypothetical protein	1.89E-04
orf19.2121_1518	3.68	ALS4	agglutinin like protein 4	8.37E-03
orf19.3441_684	3.67	FUN34	putative transporter	3.79E-03
orf19.1979_601	3.65	GIT3	glycerophosphoinositol permease	2.31E-03
orf19.6781_783	3.63		possible zinc-finger protein	1.23E-02
orf19.1097_5491	3.62	ALS4	agglutinin like protein 4	4.31E-03
orf19.6408_532	3.59	YDJ2	mitochondrial and ER import protein | dnaJ homolog	2.71E-04
orf19.2498_920	3.55	SAN1	mating-type transcriptional regulator	1.39E-04
orf19.2048_203	3.55		hypothetical protein	2.35E-05
orf19.5551_1357	3.52	MIF2	required for normal chromosome segregation and spindle integrity	7.79E-06
orf19.4590_2958	3.49	RFX1	similar to DNA-binding protein but may be missing DNA-binding domain	3.84E-05
orf19.6124_1633	3.46	ACE2	transcription activating factor	2.04E-04
orf19.3707_699	3.43	YHB1	flavohemoglobin | dihydropteridine reductase	4.88E-05
orf19.7085_1192	3.42		hypothetical protein	9.96E-05
orf19.2414_412	3.39	MPM1	mitochondrial membrane protein	7.62E-07
orf19.3113_326	3.38		conserved hypothetical protein	9.72E-04
orf19.7111.1	3.32	SOD3	superoxide dismutase	8.24E-05
orf19.4438_1074	3.31	RME1	zinc-finger transcription factor	1.98E-04
orf19.2655_652	3.31	BUB3	cell cycle arrest protein	7.43E-04
orf19.100_761	3.29	LIP11	triacylglycerol lipase	4.64E-04
orf19.3656_1108	3.29	COX15	cytochrome oxidase assembly factor	1.80E-02
orf19.6843_89	3.27		hypothetical coiled-coil protein; possible histone binding	9.82E-06
orf19.5079_3533	3.22	CDR4	ABC transporter	5.63E-06
orf19.4843_1702	3.20		conserved hypothetical protein	2.50E-03
orf19.5681_259	3.18		hypothetical protein	3.73E-03
orf19.5305_391	3.18	RHD3	conserved protein reressed in hyphal development	3.11E-04
orf19.4527_200	3.17	HGT1	hexose transporter	1.64E-02
orf19.3192_1315	3.15	STI1	heat shock protein | chaperone	5.09E-07
orf19.3122_510	3.14	ARR3	involved in arsenite transport	6.81E-05
orf19.6321_46	3.13		hypothetical protein	1.27E-06
orf19.5140_1865	3.12		hypothetical gene family	2.69E-05
orf19.3675_419	3.11	GAL7	galactose-1-phosphate uridyl transferase	9.72E-03
orf19.5961_345	3.07	NAS6	ankyrin repeat protein that interacts with the 19S regulatory particle of the 26S proteasome	2.74E-06
orf19.431_1916	3.07		potential fungal Zn(2)-Cys(6) binuclear cluster domain	1.35E-02
orf19.4372_1447	3.06		probable membrane transport protein	6.28E-05
orf19.3742_407	3.06		hypothetical protein	5.91E-06
orf19.79_1486*	3.05	ALS	cell surface agglutinin	7.74E-03
orf19.3670_890	3.03	GAL1	galactokinase	1.22E-05
orf19.6447_211	3.02	ARF1	ADP-ribosylation factor | GTP-binding protein of the ARF family	1.49E-05
orf19.742_863	3.02	ALD6	mitochondrial aldehyde dehydrogenase	2.01E-04
orf19.419_1605	3.01		hypothetical protein	1.84E-06
orf19.211_442	2.99		probable zinc finger similar to bacterial Ada DNA-protein-cysteine methyltransferase	1.27E-06
orf19.4046_148	2.96		conserved hypothetical protein	2.60E-04
orf19.2074_219	2.93		hypothetical protein	2.95E-03
orf19.3664_132	2.92	HSP31	membrane heat shock protein	1.27E-02
orf19.6997_643	2.90	FRP4	FUN34-related protein | glyoxylate pathway regulator	2.66E-04
orf19.1932_1919	2.88	FRE5	ferric reductase	2.91E-03
orf19.6489_10	2.88		conserved hypothetical protein	2.38E-03
orf19.3412_983	2.87	ATG15	lipase involved in autophagy	2.68E-04
orf19.2749_1380	2.86		conserved hypothetical protein	4.17E-03
orf19.2067_9	2.86	NFU1	nitrogen fixing protein	7.27E-05
orf19.5307_1102	2.85	JEN2	carboxylic acid transporter	5.01E-03
orf19.2125_288	2.82		hypothetical protein	1.28E-06
orf19.6594_1226	2.80	PLB3	phospholipase B	8.20E-05
orf19.85_18	2.79	GPX1	glutathione peroxidase	1.98E-04
orf19.944_973	2.79	IFG3	DAO, FAD dependent oxidoreductase | d-amino acid oxidase	8.21E-05
orf19.460_984	2.78	CEK2	serine/threonine protein kinase of MAP kinase family | Required for mating	1.41E-02
orf19.5876_56	2.76		hypothetical protein	1.62E-05
orf19.2427_4289**	2.74	POL	RNA-directed DNA polymerase	8.71E-05
orf19.2397.3	2.73		conserved hypothetical protein	1.32E-04
orf19.6964_214*	2.73	MRS107	hypothetical protein	3.28E-02
orf19.5682_213	2.68	SRP1	karyopherin-alpha or importin	7.00E-03
orf19.4970_1436	2.68		hypothetical protein	2.60E-04
orf19.847_572	2.67	YIM1	mitochondrial inner membrane protease	1.46E-04
orf19.3021_543	2.67		hypothetical protein	2.14E-03
orf19.1363_1056	2.66		conserved hypothetical protein	1.46E-04
orf19.6881_80	2.63	YTH1	cleavage and polyadenylation specificity factor	2.17E-05
orf19.7405_611	2.62		hypothetical protein	7.96E-04
orf19.4665_12	2.61		hypothetical protein	9.18E-05
orf19.4055_244	2.61		hypothetical protein	8.71E-05
orf19.1763_305	2.61	IFR1	putative reductase/dehydrogenase	8.08E-04
orf19.5672_1334	2.60	MEP2	ammonia permease	4.36E-02
orf19.1331_526	2.60	HSM3	MutS family (putative) | mismatch repair	3.02E-07
orf19.1867_739	2.58		permease of major facilitator superfamily	7.08E-04
orf19.5339_165	2.58		hypothetical protein	1.89E-04
orf19.3639_10	2.56	MAG1	3-methyladenine DNA glycosylase	1.63E-03
orf19.6301_92*	2.56		hypothetical protein	8.57E-06
orf19.5751_218	2.54	ORM1	involved in response to unfolded proteins	4.17E-05
orf19.1606_681	2.54		hypothetical protein	1.05E-03
orf19.6248_247	2.53		hypothetical protein	3.11E-04
orf19.2218_258	2.53		hypothetical protein (merged with orf.1861)	1.20E-03
orf19.4411_744	2.53	HOS1	histone deacetylase	6.14E-03
orf19.733_312	2.53		conserved hypothetical protein	5.56E-03
orf19.4982_1561	2.53	TGL3	triglyceride lipase-cholesterol esterase	2.13E-03
orf19.4413_13	2.53	CMD1	calmodulin	2.12E-03
orf19.5569_1850	2.51	SRC1	Spliced mRNA and Cell cycle regulated gene	4.92E-04
orf19.5457_12	2.51		conserved hypothetical protein	6.55E-03
orf19.2467_688	2.50	PRN1	RNA pol II transcription cofactor	6.20E-05
orf19.7091_262	2.49		conserved hypothetical protein	1.79E-05
orf19.6747_221	2.48		conserved hypothetical protein	3.21E-04
orf19.31_168	2.48	CIS35	potential cell wall protein | member of a group of C.albicans orfs that are weakly similar to Sc CIS3/PIR3/PIR1	2.92E-03
orf19.2367_180	2.48		conserved hypothetical protein	4.99E-03
orf19.5525_242	2.48		conserved hypothetical protein	4.18E-04
orf19.2398_149	2.46		hypothetical protein	1.37E-03
orf19.1815_514	2.46	TIF6	translation initiation factor 6 (eIF6)	1.49E-05
orf19.2046_508	2.46	POT13	acetyl-CoA C-acyltransferase, peroxisomal | fatty acid beta-oxidation	1.10E-02
orf19.4035_387	2.46	GAS1	GPI anchored surface protein	6.75E-04
orf19.7115_873	2.45	SAC7	GTPase activating protein (GAP) for RHO	1.89E-04
orf19.3407_554	2.45	RAD18	DNA repair protein and ATPase	2.50E-02
orf19.3586_0	2.45		conserved hypothetical protein	4.31E-03
orf19.1617_181	2.45		conserved hypothetical protein	3.97E-02
orf19.4337_1791	2.44	ESBP6	monocarboxylate permease	6.88E-04
orf19.3672_1902	2.44	GAL10	UDP glucose-4-epimerase	8.25E-04
orf19.3845_47	2.43		zinc finger protein	3.40E-04
orf19.22_458	2.43		MPV17 homolog | hypothetical protein	2.14E-03
orf19.7436_1378	2.43	ADF1	adhesion and aggregation mediating surface antigen	6.18E-07
orf19.6963_221*	2.42	MRS107	hypothetical protein	2.40E-04
orf19.449_1117	2.42		predicted phosphatidyl synthase	1.68E-04
orf19.6324_477	2.42	VID27	vacuole import and degradation	2.10E-03
orf19.2942_1260	2.42	DIP52	dicarboxylic amino acid permease	2.38E-04
orf19.6957.3*	2.42		hypothetical protein with homology to part of Isocitrate dehydrogenase (NAD+) subunit 1	2.01E-02
orf19.5956_24	2.40	PIN3	SH3 domain protein	2.25E-04
orf19.7227_89	2.40		conserved hypothetical protein	2.34E-02
orf19.5159_598	2.40		conserved hypothetical protein	8.31E-04
orf19.4783_1356	2.40		conserved hypothetical protein	1.40E-02
orf19.1911_237	2.39	TOS2	Target of SBF	4.12E-03
orf19.3526_1269	2.39	ITR2	myo-inositol transporter	6.80E-03
orf19.2463_694	2.38	PRN2	RNA pol II transcription cofactor	1.59E-03
orf19.4048_532	2.38	DES1	probable fatty acid desaturase	3.37E-05
orf19.7325_169	2.38	SCO1	inner mitochondrial membrane protein	1.07E-03
orf19.5749_1	2.37	SBA1	HSP90 associated co-chaperone	6.40E-04
orf19.1048_733	2.37	IFD1	conserved aryl-alcohol dehydrogenase	1.26E-04
orf19.874_202	2.37		conserved hypothetical protein	2.32E-02
orf19.5911_81	2.36	CMK1	Ca2+/calmodulin-dependent protein kinase	7.81E-04
orf19.4720_41	2.35	CTR2	copper transpport protein	2.83E-03
orf19.814_1972	2.34	SSY1.5	transcriptional regulator of multiple amino acid permeases	3.03E-06
orf19.7003_265*	2.34		hypothetical protein	2.98E-03
orf19.6113_244	2.34		hypothetical protein	2.56E-03
orf19.5069_154	2.34		conserved hypothetical protein	4.53E-05
orf19.2803_82	2.33	HEM13	coprophyrinogen oxidase | heme biosynthesis	1.60E-03
orf19.7450_648	2.33	BNI5	may localize to mother-bud neck in a septin-dependent manner | similar to mammalian homer porteins	2.15E-03
orf19.5170_877	2.32	ENA2	P-type ATPase involved in Na+ efflux	2.51E-02
orf19.1861_65	2.32		SH3 domains protein (merged with orf19.2218)	2.91E-03
orf19.393_61	2.32	APS3	AP-3 complex subunit functioning in gogi-to-vacuole protein transport	1.92E-07
orf19.878_54	2.31	YNG2	NuA4 histone acetyltransferase complex component	3.77E-03
orf19.4155.12*	2.30		similar to protion of isocitrate dehydrogenase 1 alpha-4-beta-4 subunit	1.26E-02
orf19.6487_337	2.30		hypothetical protein	8.18E-04
orf19.2568_179	2.29	WWM1	involvd in response to dessication	5.60E-06
orf19.5459_51	2.29	PBP1	poly(A)-binding protein binding protein	6.49E-03
orf19.5686_374	2.29		hypothetical protein	1.56E-04
orf19.3674_835	2.29	GAL102	UDP-glucose 4-epimerase	5.70E-03
orf19.882_1800	2.28	HSP78	heat shock protein of clpb family of ATP-dependent proteases	1.73E-06
orf19.2610_159	2.27	ARC2	protein with specific affinity for G4 quadruplex nucleic acids	1.97E-04
orf19.2832_1864	2.25		conserved hypothetical protein	1.55E-02
orf19.2580_668	2.25	HST2	similar to Hst1p and Sir2p putative histone deacetylases	1.68E-02
orf19.5741_2774	2.25	ALS1-1	agglutinin like protein 1	6.79E-03
orf19.2863.1	2.24	ERV1	sulfhydryl oxidase	7.34E-05
orf19.3923_505	2.24		conserved hypothetical protein	5.69E-04
orf19.3858_286	2.24		hypothetical protein	1.07E-03
orf19.1607_2114	2.24	ALR1	putative divalent cation transporter	3.52E-05
orf19.5920_253	2.24		hypothetical protein	8.91E-05
orf19.7078_113	2.24		conserved hypothetical protein	9.69E-07
orf19.7267_27	2.23		conserved hypothetical protein	3.16E-05
orf19.3499_423	2.23		hypothetical protein	4.67E-03
orf19.4555_246	2.22	ALS4	agglutinin-like protein 4	2.32E-02
orf19.5394.1	2.22	PET191	mitochondrial regulator	2.68E-02
orf19.5291_552	2.22	SCS3	inositol phospolipid biosynthesis	3.94E-04
orf19.413.1	2.21	RPS27A	ribosomal protein S27A	2.91E-04
orf19.4622_305	2.21		hypothetical protein	1.39E-02
orf19.6070_963	2.21	ENA5	Na+ ATPase	4.87E-02
orf19.6451_235**	2.21	POL99	pol polyprotein	3.80E-03
orf19.1488_22	2.21		hypothetical protein	4.06E-03
orf19.6102_612	2.21	CST6	ATF/CREB activator	5.39E-03
orf19.2006.1	2.21	COX17	cytochrome c oxidase copper chaperone	2.56E-04
orf19.4869_1197	2.21	SFU1	GATA type transcriptional activator of nitrogen-regulated genes	4.74E-05
orf19.5640_1494	2.21	PEX5	peroxisomal protein receptor	1.76E-03
orf19.4546_1146	2.21	HOL4	member of major facilitator superfamily multidrug-resistance protein	2.55E-02
orf19.7544_44	2.20	CTA2	transcriptional activation	1.31E-03
orf19.6614_3186	2.20		DEAD/DEAH box helicase	2.70E-02
orf19.2303_508	2.20		conserved hypothetical protein	2.32E-02
orf19.7250_305	2.20		conserved hypothetical protein	9.47E-05
orf19.4177_401	2.19	HIS5	histidinol-phosphate aminotransferase	3.98E-03
orf19.1407_952	2.19		conserved hypothetical membrane protein	3.58E-02
orf19.6048_184	2.19	PMT3	mannosyltransferase	3.71E-03
orf19.1187_1941	2.19	CPH2	bHLH DNA-binding protein that promotes hyphal development	8.92E-07
orf19.3713_466	2.18		hypothetical protein	6.50E-03
orf19.6554_180	2.18		conserved hypothetical protein	4.36E-04
orf19.171_1445	2.18	DBP2	DEAD box RNA helicase	5.61E-03
orf19.1623_867	2.18	CAP1	transcriptional activator involved in oxidative stress response	2.64E-05
orf19.42_308*	2.18		transport protein	5.16E-03
orf19.4436_35	2.17	GPX2	glutathione peroxidase	7.12E-04
orf19.7676_924	2.17	SOR1	sorbitol dehydrogenase	1.82E-04
orf19.1416_0	2.17	COX11	cytochrome-c oxidase assembly protein	1.42E-03
orf19.5463_771	2.16	SEC6	exocyst complex subunit	3.31E-02
orf19.4031_1433	2.16		conserved hypothetical protein	4.37E-04
orf19.5823_188	2.16	SGT2	small glutamine-rich tetratricopeptide repeat containing protein | similarity to protein phosphatases	9.41E-03
orf19.2030_124	2.16		hypothetical protein	9.32E-03
orf19.2049_624	2.16		hypothetical protein	4.69E-05
orf19.1925_42	2.15	CTA2-10	transcription factor	4.66E-03
orf19.1034_94	2.15	ATM2	putative steroid binding	7.96E-03
orf19.409_86	2.14		conserved hypothetical protein	1.43E-04
orf19.3342_1665	2.14		hypothetical protein	7.13E-04
orf19.1453_1564	2.14	SPT5	transcription elongation factor	8.99E-03
orf19.3004_764	2.14		conserved hypothetical protein	3.84E-03
orf19.3471_112	2.13		hypothetical protein	1.45E-03
orf19.2105_550	2.13	CWC24	zinc finger protein	5.79E-04
orf19.5094_1885	2.12	BUL3	ubiquitin-mediated protein degradation	5.01E-04
orf19.2342_545	2.12	SFT2	similar to mammalian syntaxin 5	1.98E-06
orf19.2848_1810	2.12	APG13	involved in autophagy	1.08E-04
orf19.1486_190	2.12		hypothetical protein	2.73E-06
orf19.699_279	2.11		hypothetical protein	1.56E-03
orf19.3323_686	2.11		hypothetical protein	4.58E-05
orf19.5785_401	2.11		hypothetical protein	4.93E-03
orf19.3618_1190	2.11	YWP1	putative cell wall protein	3.68E-04
orf19.4054_25	2.10	CTA2	transcriptional regulation	1.87E-04
orf19.2179_1006	2.10	ARN1	iron-siderophore transporter	5.94E-03
orf19.2107.1	2.10	STF2	ATP synthase regulatory factor	5.61E-07
orf19.3874_1600	2.10		hypothetical protein	5.03E-04
orf19.203_1031	2.09	STB3	Sin3p binding protein	2.14E-03
orf19.6674_771	2.08	BTS1	geranylgeranyl diphosphate synthase	5.81E-05
orf19.7644_196	2.08	APC11	ubiquitin-protein ligase; Anaphase Promoting Complex	8.42E-04
orf19.4740_167	2.08	PRH1	peptidyl-tRNA hydrolase	7.94E-03
orf19.5192_1*	2.08		conserved hypothetical protein	2.86E-02
orf19.5133_2470	2.08		hypothetical DNA binding protein	3.58E-03
orf19.7519_168	2.08		hypothetical protein	1.63E-03
orf19.5165_1045	2.07		conserved hypothetical protein	2.82E-03
orf19.5337_449	2.07	UBC15	E2 ubiquitin conjugating enzyme	1.69E-03
orf19.6387_2494	2.06	HSP104	heat shock protein 104	5.04E-03
orf19.1014_291*	2.06		probable 26S proteasome regulatory subunit	5.05E-04
orf19.2616_4105	2.06	ATG26	UDP-glucose:sterol glucosyltransferase	1.32E-02
orf19.6993_1316	2.06	GAP2	general amino acid permease	2.13E-03
orf19.5775.3*	2.05		isocitrate dehydrogenase (NAD+) subunit 1	5.95E-03
orf19.5752_1052	2.05		conserved hypothetical protein	1.33E-04
orf19.2098_693	2.05	ARO8	aromatic amino acid aminotransferase	7.53E-04
orf19.675_241	2.05		hypothetical protein	2.98E-03
orf19.3089_329	2.05		possibly involved in intramitochondrial sorting	1.04E-03
orf19.6139_1376	2.05	FRE7	ferric reductase	1.05E-04
orf19.6191_51	2.04	CTA2	transcriptional activator	2.99E-02
orf19.250_750	2.04	SLC1	fatty acyltransferase	3.02E-02
orf19.3073_270	2.04		hypothetical protein	1.48E-03
orf19.7125_731	2.04		hypothetical protein	3.09E-02
orf19.3124_254	2.04	MAP1	methionine aminopeptidase	1.57E-03
orf19.1744_726	2.04	HEM4	uroporphyrinogen III synthase | heme biosynthesis	1.67E-02
orf19.6811_133	2.03	ISA2	mitochondrial protein required for iron metabolism	9.97E-05
orf19.399_1354	2.03	YPK2	ser/thr protein kinase	3.07E-04
orf19.2607_135	2.03	PMU2	phosphomutase homolog	5.16E-03
orf19.6112_54	2.03	CTA2	putative transcriptional activator	2.34E-03
orf19.3475_329	2.02		Gag protein	1.50E-06
orf19.183_177	2.02	HIS3	imidazoleglycerol-phosphate dehydratase	1.28E-02
orf19.6180_79	2.02		conserved hypothetical protein	1.33E-02
orf19.4706_128	2.02		low similarity to prion protein	1.29E-02
orf19.1281_356	2.01		conserved hypothetical protein	7.32E-04
orf19.5114_59	2.01	GRD19	retrieval from vacuole to Golgi	2.15E-02
orf19.441_313	2.01	RPT1	26S protease subunit component | ATPase | Required for degradation of ubiquitinated substrates and for anaphase chromosome separation	4.70E-03
orf19.4943_1228	2.01	PSA2	mannose-1-phosphate guanyltransferase	2.79E-03
orf19.2333_1339	2.01		highly conserved oxidoreductase	1.93E-02
orf19.5251_2284	2.00		potential fungal Zn(2)-Cys(6) binuclear cluster domain	5.28E-02
DOWNREGULATED GENES				
orf19.6821_2288	0.50	APC2	subunit of the Anaphase Promoting Complex	3.79E-03
orf19.3247_6372	0.50		highly conserved hypothetical protein	7.61E-04
orf19.4591_1781	0.50	CAT2	carnitine acetyltransferase	1.63E-04
orf19.5943_1094	0.50		conserved hypothetical protein	1.03E-02
orf19.4594_512	0.50	CLC1	clathrin light chain	1.05E-05
orf19.2896_599	0.50	SOU1	peroxisomal 2,4- dienoyl-CoA reductase, and sorbitol utilization protein	4.59E-04
orf19.7354_747	0.49	LAC2	longevity-assurance protein	1.06E-02
orf19.479.2	0.49	SEC22	ER to Golgi protein transport synaptobrevin (V-SNARE)	6.77E-05
orf19.6796_414	0.49	YSA1	sugar-nucleotide hydrolase	7.54E-03
orf19.5968_133	0.49	RDI1	Rho GDP dissociation inhibitor	2.05E-06
orf19.3577.1	0.49		conserved hypothetical protein	3.73E-02
orf19.4675_1643	0.49		conserved hypothetical protein	1.61E-02
orf19.6689_654	0.49	ARG4	argininosuccinate lyase	1.54E-03
orf19.2533.1	0.49	SBH1	Sec61p-Sss1p-Sbh1p complex component, involved in protein translocation into the endoplasmic reticulum	1.37E-05
orf19.1797_497	0.49		conserved hypothetical protein	1.11E-03
orf19.1598_1274	0.49	ERG24	sterol C-14 reductase	1.68E-04
orf19.2021_492	0.49	HXT5	hexose transporter	1.27E-03
orf19.3063_215	0.49	DPB3	DNA-directed DNA polymerase epsilon, subunit C	2.87E-04
orf19.5065_999	0.49	ERD1	required for retention of luminal ER proteins	2.09E-02
orf19.2298_1199	0.49	WBP1	oligosaccharyl transferase beta subunit precursor	8.83E-05
orf19.3649_652	0.48	FES1	adenyl-nucleotide exchange factor activity	9.66E-04
orf19.868_1341	0.48		putative adenosine deaminase | transcriptional regulation	7.86E-08
orf19.5648_471	0.48		putative nuclear export factor	2.35E-03
orf19.2341_145	0.48	HNT1	similarity to protein kinase C inhibitor-I, histidine triad nucleotide-binding proteins	4.48E-04
orf19.4733_749	0.48	YMC3	mitochondrial carrier protein	8.89E-04
orf19.1492_1874	0.48	PRP39	pre-mRNA splicing factor | U1 snRNP protein	6.13E-03
orf19.2446_359	0.48		highly conserved hypothetical protein	6.79E-04
orf19.1278_139	0.48		conserved hypothetical protein	9.74E-04
orf19.3607_1112	0.48		alpha/beta hydrolase	1.48E-03
orf19.1960_1314	0.48	CLN3	G1 cyclin	4.17E-03
orf19.6769_1990	0.48		conserved hypothetical protein	5.70E-04
orf19.254_859	0.48		hypothetical protein	1.67E-04
orf19.3669_1723	0.48	SKS1	serine/threonine protein kinase	8.40E-04
orf19.6968_2365	0.48		conserved hypothetical protein	8.21E-04
orf19.1631_945	0.47	ERG6	S-adenosyl-methionine delta-24- sterol-c-methyltransferase	1.39E-03
orf19.6893_888	0.47	RUD3.3	relieves uso1-1 transport defect | golgin-160 related protein	8.58E-03
orf19.873_83	0.47		hypothetical protein	4.35E-03
orf19.3633_410	0.47		transthyretin precursor (Prealbumin)	1.51E-02
orf19.7593_1317	0.47	ASP1	L-asparaginase	9.54E-05
orf19.6864_63	0.47		conserved hypothetical protein	9.27E-03
orf19.2836_392	0.47		conserved hypothetical protein	1.64E-02
orf19.6624_1111	0.47		TBC domain protein	3.28E-02
orf19.1390_1043	0.47	PMI1	mannose-6-phosphate isomerase	4.31E-03
orf19.3394_506	0.46		hypothetical protein	6.69E-03
orf19.7409_568	0.46	ERV25	component of COPII coat of ER- derived vesicles | p24 protein family	6.24E-05
orf19.3417_2120	0.46	ACF2	endo-1,3-beta- glucanase, and involved in actin polymerization	4.43E-02
orf19.4197_756	0.46	YHM2	DNA binding protein | mtDNA stabilizing protein | mitochondrial inner membrane protein	1.38E-02
orf19.568_915	0.46	SPE2	S-adenosylmethionine decarboxylase	1.73E-05
orf19.2636_205	0.46		conserved hypothetical protein	1.27E-02
orf19.7016_1640	0.46		vacuolar endopolyphosphatase	2.10E-02
orf19.1190_2478	0.46	VPH3	vacuolar ATPase V0 domain subunit a	4.38E-05
orf19.5112_1741	0.46	TKL1	transketolase 1	2.31E-04
orf19.6286_512	0.46		conserved hypothetical protein	1.53E-03
orf19.3839_587	0.45	SAP10	secretory aspartyl proteinase	1.31E-02
orf19.2087_989	0.45	SAS2	zinc finger protein involved in silencing HMR	1.62E-03
orf19.3221_3206	0.45	CPA2	carbamoyl phosphate synthetase large subunit, arginine biosynthesis	9.53E-05
orf19.4825_149	0.45	FMC1	formation of mitochondrial complexes | assembly factor of ATP synthase in heat stress | Formation of Mitochondrial Cytochromes	5.79E-03
orf19.2842_1951	0.45	GZF3	transcriptional repressor similar to zinc finger Dal80	2.79E-04
orf19.6134_2330	0.45		conserved hypothetical protein	1.17E-05
orf19.4900_2286	0.45	MNN13	mannosyltransferase	1.31E-03
orf19.6291_2766	0.45	FUN30	helicase of the Snf2/Rad54 family	5.87E-04
orf19.92_2412	0.45		conserved hypothetical protein	2.71E-03
orf19.4870_1388	0.45	DBP3	ATP-dependent RNA helicase CA3 of the DEAD/DEAH box family	1.14E-02
orf19.4624_1202	0.45	HRT2	Ty3 transposition effector	4.07E-03
orf19.4229_107	0.45	DDP1	polyphosphate phosphohydrolase	1.77E-04
orf19.7321_1583	0.45		conserved hypothetical protein	1.96E-02
orf19.6318_217	0.45		conserved hypothetical protein	3.05E-07
orf19.3065_712	0.44	DAO1	D-amino acid oxidase	8.58E-04
orf19.4056_988	0.44		GATA-family DNA binding proteins	1.87E-02
orf19.2170_2566	0.44		membrane transporter	4.56E-04
orf19.1670_2527	0.44	BRO1	involved in integral membrane protein trafficking	4.23E-03
orf19.5628_801	0.44	DIC1	mitochondrial dicarboxylate transport protein	6.50E-04
orf19.290_4218	0.44	KRE5	UDPglucose- glycoprotein glucose phosphotransferase	9.55E-05
orf19.5231.2	0.44	ATP19	subunit K of mitochondrial ATP Synthase	1.43E-05
orf19.4699_1941	0.44		conserved hypothetical membrane protein	9.75E-05
orf19.2846_312	0.44		hypothetical protein	4.16E-04
orf19.1107_119	0.44		conserved hypothetical protein	7.24E-05
orf19.4236_1587	0.43	RET2	coatomer (COPI) complex delta subunit	1.06E-04
orf19.5437_488	0.43	GPP1	DL-glycerol-3-phosphatase	4.47E-04
orf19.1761_264	0.43	OST2	oligosaccharyltransferase epsilon subunit	2.92E-05
orf19.5171_2330	0.43	PMT1	mannosyltransferase	5.99E-05
orf19.6627_482	0.43		retrovirus-related like polyprotein	5.63E-04
orf19.6699_755	0.43	HIS2	histidinolphosphatase	2.16E-02
orf19.1092_1475	0.43	RHK1	dol-p-man dependent alpha(1-3) mannosyltransferase	1.54E-03
orf19.4600.1	0.43	DPM3	dolichol-phosphate-mannose synthase	5.96E-07
orf19.7479_2570	0.43	NTH1	neutral trehalase (alpha,alpha-trehalase)	2.62E-04
orf19.1427_1347	0.43		conserved hypothetical transporter	4.04E-04
orf19.5851_2414	0.43	STE13	dipeptidyl aminopeptidase	4.49E-04
orf19.1306_742	0.42		conserved oxidase	1.01E-03
orf19.1963_1144	0.42	GDS1	involved in nuclear control of mitochondria	1.21E-02
orf19.4000_1818	0.42	PHO2	homeobox transcription factor, positive regulator of PHO5 and other genes	5.36E-03
orf19.2671_1046	0.42	NDI1	NADH dehydrogenase	4.67E-02
orf19.4099_2254	0.42	ECM17	extracellular sulfite reductase	7.30E-04
orf19.3873_1029	0.42	ARC40	component of the ARP2-3 complex	9.84E-06
orf19.4755_2734	0.42	KEX2	Kexin protease | late Golgi endoprotease that processes of alpha-factor	2.10E-03
orf19.732_60	0.42	SPS22	carbonyl reductase similar to SOU1 and SOU2	2.87E-02
orf19.2822_41	0.42		hypothetical protein	4.35E-03
orf19.3547_1916	0.42	PUF6	member of the PUF protein family	5.97E-04
orf19.4477_551	0.41	IFD4	aryl-alcohol dehydrogenase	5.60E-03
orf19.3133_1848	0.41	GUT2	mitochondrial glycerol-3-phosphate dehydrogenase	5.69E-05
orf19.3836_405	0.41		conserved hypothetical protein	1.71E-04
orf19.4440_2241	0.41	COG3	Conserved Oligomeric Golgi complex 3 secretion (golgi retention) deficient | required for vesicle tethering to the yeast Golgi apparatus	1.26E-02
orf19.6008_2496	0.41		conserved hypothetical protein	2.31E-03
orf19.7328_2563	0.41	CAP100	Candida albicans p100 homolog	9.47E-04
orf19.6818_3344	0.41		RNA helicase	2.00E-03
orf19.2805_2280	0.41	PEX99	putative peroxisomal protein	1.33E-03
orf19.4445_1331	0.40		hypothetical protein	1.27E-02
orf19.1012_357	0.40	APS1	AP-1 clathrin associated protein complex subunit	1.84E-06
orf19.3740_692	0.40		hypothetical protein	9.11E-03
orf19.3181.1	0.40	NCE11	involved in non-classical protein export pathway	8.11E-06
orf19.5438_160	0.40		hypothetical protein	3.79E-05
orf19.4479_1735	0.40		conserved hypothetical protein	1.99E-03
orf19.4579_799	0.39	ERV29	ER-Golgi transport vesicle protein	4.04E-03
orf19.5025_1446	0.39	MET3	ATP sulfurylase, Amino acid metabolism	6.42E-05
orf19.1985_249	0.39		conserved hypothetical protein (*merged with orf19.3488*)	1.10E-04
orf19.3335_444	0.39		hypothetical protein	2.30E-03
orf19.3459_1014	0.39	MCK1	serine/threonine/tyrosine protein kinase involved in chromosome segregation	1.05E-02
orf19.2724_1039	0.39		hypothetical protein	2.82E-03
orf19.5753_1345	0.39	STL1	sugar transporter	2.32E-04
orf19.3573_3084	0.39	PEX6	peroxisomal assembly protein | AAA ATPase	2.17E-03
orf19.3507_322	0.39	MCR1	cytochrome b5 reductase	6.16E-05
orf19.5462_410	0.39		hypothetical protein	4.70E-03
orf19.1719_1613	0.39	SGA1	glucoamylase	1.15E-02
orf19.5777_544	0.38		involved in pseudohyphal growth, resistance to NaCl and H2O2	1.26E-05
orf19.1203.1	0.38		conserved hypothetical protein	9.57E-05
orf19.3226_19	0.38	NPC2	vacuolar protein and homolog of Niemann Pick type C protein	2.74E-03
orf19.2837_903	0.38	ALG5	UDP-glucose:dolichyl-phosphate glucosyltransferase	1.25E-02
orf19.398_236	0.38		hypothetical protein	3.18E-03
orf19.6985_2614	0.37	TEA1	transcription factor with fungal Zn(2)- Cys(6) binuclear cluster domain | TY1 enhancer activator	2.07E-02
orf19.889_1175	0.37	THI20	thiamine biosynthesis | phosphomethylpyrimidine kinase	5.51E-03
orf19.2416.1	0.37	MLC1	myosin light chain	3.28E-05
orf19.10_1251	0.37	ALK8	cytochrome p450	4.49E-03
orf19.6527_3245	0.37	PRM10	pheromone-regulated membrane	7.68E-03
orf19.1344_53	0.36		hypothetical protein	2.65E-02
orf19.3041_1842	0.36		conserved hypothetical protein with similarity to ROD1	4.46E-04
orf19.6196_170	0.36		hypothetical protein	1.71E-04
orf19.1495_650	0.36	UTR4	hydrolase	1.72E-02
orf19.4886_253	0.35		hypothetical protein	1.33E-04
orf19.1066_75	0.35		conserved hypothetical protein	1.12E-04
orf19.2897_637	0.35	SOU2	peroxisomal 2,4- dienoyl-CoA reductase and sorbitol utilization protein	4.59E-02
orf19.677_658	0.35	CHO1	phosphatidylserine synthase	2.68E-05
orf19.3969_1973	0.35	HSR1	heat-shock related protein	1.92E-05
orf19.3994_956	0.35	OST3	oligosaccharyltransferase gamma subunit	1.64E-02
orf19.7330_45	0.35	PET18	transcriptional regulator	6.36E-04
orf19.3782_1583	0.35		acetyl-coenzyme A transporter	8.09E-03
orf19.946_272	0.35	MET14	adenylylsulfate kinase	7.23E-04
orf19.5295_1010	0.34		conserved hypothetical protein	6.58E-03
orf19.94_365	0.34		hypothetical protein	1.47E-03
orf19.4264_681	0.33		hypothetical protein	2.05E-05
orf19.535_248	0.33		hypothetical serine-rich protein	3.69E-06
orf19.6988_922	0.33	OST1	oligosaccharyltransferase | involved in glycosylation in the ER lumen	1.01E-07
orf19.3469_1051	0.33		hypothetical protein	2.99E-04
orf19.3520_80	0.33		hypothetical protein	4.36E-03
orf19.4903_968	0.33	GPI12	N-acetylglucosaminylphosphatidylinositol de-N-acetylase	2.33E-02
orf19.4076_3165	0.33	MET10	sulfite reductase flavin-binding subunit	4.11E-06
orf19.1946_664	0.32		conserved hypothetical protein	1.02E-04
orf19.334_252	0.32		hypothetical protein	1.01E-05
orf19.3016_346	0.32		conserved hypothetical protein	5.25E-03
orf19.3374_455	0.31	ECE1	secreted cell elongation protein	3.44E-03
orf19.1120_153	0.30		hypothetical protein	3.84E-03
orf19.2269_481	0.30		3-phosphoserine phosphatase	2.48E-02
orf19.3488_677	0.30		hypothetical protein (merged with orf19.1985)	2.97E-02
orf19.691_1048	0.29	GPD1	glycerol-3-phosphate dehydrogenase	4.31E-04
orf19.5517_879	0.29	ADH6	alcohol dehydrogenase	4.12E-04
orf19.3419_687	0.29	MAE1	mitochondrial malate dehydrogenase	3.16E-04
orf19.242.2	0.27	YSY6	secretory pathway protein	2.89E-06
orf19.7411_204	0.26	OAC1	mitochondrial oxaloacetate transport protein	2.83E-03
orf19.1112_2071	0.26	BUD7	involved in bud-site selection	3.32E-06
orf19.7324_806	0.25	THI13	pyrimidine precursor biosynthesis enzyme	6.80E-05
orf19.5557_2117	0.24	MNN43	transfer mannosylphosphate to oligosaccharides	3.37E-03
orf19.5992_1255	0.22		zinc finger transcription factor	3.07E-04
orf19.5210_1072	0.21	XBP1	transcription factor	1.18E-03
orf19.2552_2609	0.20	PMR2	Ca2+ ATPase	2.20E-08
orf19.2038_882	0.19		hypothetical protein	6.33E-04

* Deleted in the CGD assembly 21,

** Deleted Tn element in CGD assembly 21

**Figure 1:**
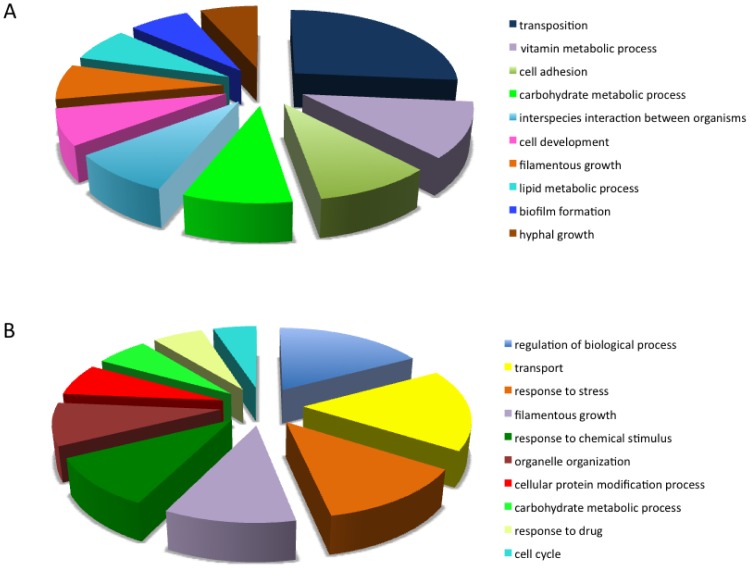
Ten most represented functional categories affected by growth of *C. albicans* in spaceflight conditions. The top ten of functional categories was determined by calculating (A) the ratio of the number of genes in category X to the total number of genes in the genome assigned to category X, and (B) the ratio of the number of genes in category X to the total number of genes differentially regulated by spaceflight.

In order to evaluate global, high-level changes in gene expression, differentially expressed genes were classified into Biological Process categories (**Table 3**), using GO Slim Mapper (September 12, 2013 version) [50]. While the function of many of the differentially regulated genes is currently unknown (not included in **Table 3** and **Figure 1**), several categories of interest were found (**Table 3**). Differentially expressed genes are presented in **Table 3** as (i) the ratio of the number of genes in category X to the total number of genes in the genome assigned to category X, and (ii) the ratio of the number of genes in category X to the total number of genes differentially regulated by spaceflight. This classification indicated that spaceflight affects a broad range of cellular functions, ranging from biofilm formation to vesicle-mediated transport. It is worth noting that many genes are assigned to more than one category; therefore, the sum totals of the columns in **Table 3** do not equal either the total number of genes in the genome or 100%.

**Table 3:** Biological process categories of *C. albicans* affected by spaceflight conditions as compared to ground control, based on GO Slim Mapper analysis.

GO term	# Genes in genome assigned (A)	# Genes differentially regulated (B)	Percentage of # genes in genome (A/B)	Percentage of # genes differentially regulated (B/454)*
biofilm formation	54	12	22.2%	2.6%
carbohydrate metabolic process	241	29	12.0%	6.4%
cell adhesion	45	7	15.6%	1.5%
cell budding	84	3	3.6%	0.7%
cell cycle	366	24	6.6%	5.3%
cell development	82	10	12.2%	2.2%
cell wall organization	155	12	7.7%	2.6%
cellular homeostasis	130	12	9.2%	2.6%
cellular membrane organization	212	9	4.2%	2.0%
cellular protein modification process	471	32	6.8%	7.0%
cellular respiration	105	4	3.8%	0.9%
conjugation	93	7	7.5%	1.5%
cytokinesis	117	4	3.4%	0.9%
cytoskeleton organization	177	9	5.1%	2.0%
DNA metabolic process	307	19	6.2%	4.2%
filamentous growth	511	51	10.0%	11.2%
generation of precursor metabolites and energy	167	7	4.2%	1.5%
growth of unicellular organism as a thread of attached cells	78	6	7.7%	1.3%
hyphal growth	181	5	2.8%	1.1%
interspecies interaction between organisms	106	14	13.2%	3.1%
lipid metabolic process	251	23	9.2%	5.1%
nucleus organization	47	1	2.1%	0.2%
organelle organization	838	42	5.0%	9.3%
pathogenesis	352	15	4.3%	3.3%
protein catabolic process	152	10	6.6%	2.2%
protein folding	80	5	6.3%	1.1%
pseudohyphal growth	52	2	3.8%	0.4%
regulation of biological process	1356	82	6.0%	18.1%
response to chemical stimulus	612	49	8.0%	10.8%
response to drug	399	28	7.0%	6.2%
response to stress	504	61	12.1%	13.4%
ribosome biogenesis	286	6	2.1%	1.3%
RNA metabolic process	669	17	2.5%	3.7%
signal transduction	189	5	2.6%	1.1%
translation	387	2	0.5%	0.4%
transport	951	81	8.5%	17.8%
transposition	4	1	25.0%	0.2%
vesicle-mediated transport	288	20	6.9%	4.4%
vitamin metabolic process	59	5	8.5%	1.1%

*Based on 454 genes differentially regulated in response to spaceflight

The ten functional categories with the greatest number of differentially expressed genes in response to spaceflight expressed as a percent of assigned genes in the genome (**Figure 1A**) and/or the total number of differentially regulated genes (**Figure 1B**) include biofilm formation, cell adhesion, transport, interspecies interaction, response to chemical stimulus, response to stress, response to drugs, carbohydrate metabolism, and filamentous growth (**Table 3** **,** **Figure 1**).

**Figure 2:**
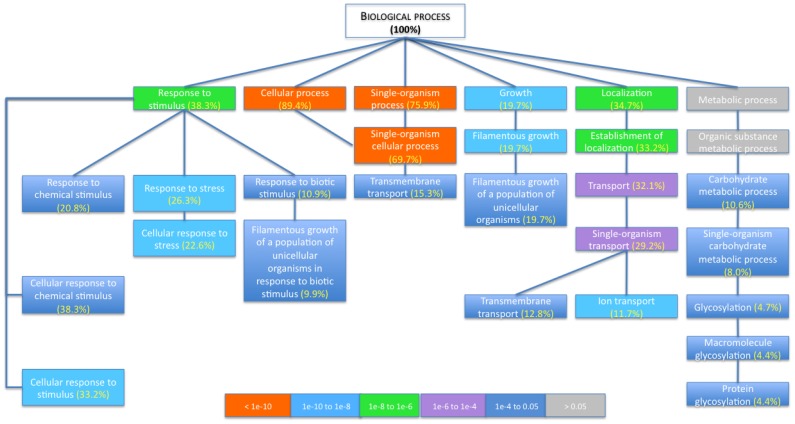
Hierarchical ranking of the GO Term Finder Process categories that were significantly enriched. Only categories that are significantly enriched (p<0.05) are presented, except for those labeled grey added for hierarchical purposes. Subcategories with more than 2 higher rank categories that were not significantly enriched are not included in this figure (i.e., dicarboxylic acid transport and copper ion transport). For clarity purposes, categories with more than one connector are not presented, if the connecting category/categories was/were not significantly enriched. Color codes indicate p-values.

Next, we analyzed whether specific biological processes within our data set were significantly enriched, using GO Term Finder. **Figure 2** presents the hierarchical ranking of the GO Term Finder Process categories that were significantly enriched (p<0.05). These categories include filamentous growth, carbohydrate metabolism, response to chemical stimulus, response to stress, and transport; which were also represented in the top ten categories identified with GO Slim Mapper (**Table 3**, **Figure 1**).

Categories that were significantly enriched by spaceflight culture (**Figure 2**) and are of particular interest for this study given their direct role in the infectious disease process are response to stress and filamentation. In addition, we were interested in differentially regulated genes involved in (i) biofilm formation, cell aggregation, and random budding given our phenotypic observations (described below), and (ii) response to drugs and RNA binding given previous findings from *C. albicans* and other microbial pathogens cultured in spaceflight and/or spaceflight-analogue culture systems [11], [12], [35]. These specific categories are analyzed in greater detail below. While these categories were initially identified using the set criteria of significance (p<0.05, fold-change >2), the number of genes belonging to pathways within these specific targeted categories of interest was enlarged using less stringent criteria (p<0.07 or fold-change >1.5, indicated with †).

To validate the microarray data, qRT-PCR analysis of a targeted selection of genes that were differentially regulated with microarray was performed. Expression of the target genes (*ALS1*, *CAP1*, *ERG6*, *YTH1*, *HSP31*, *GPX2*) was normalized using the averaged expression of four housekeeping genes (*ACT1*, *PMA1*, *RIP*, *RPP2B*) [52]. All analyzed genes were found differentially regulated with qRT-PCR in the same direction as found with microarray analysis, and for four out of six analyzed genes, the differential regulation was significant (p<0.05 or p<0.01) (**Table 4**).

**Table 4:** Relative gene expression of *C. albicans* grown in spaceflight versus ground control conditions, as determined by microarray and qRT-PCR analysis.

Gene	Category	Fold-change microarray	Fold-change qRT-PCR
*ALS1*	Biofilm	2.25**	1.83*
*CAP1*	Transcriptional regulator	2.18**	3.39**
*ERG6*	Antifungal drug resistance	0.48**	0.46
*YTH1*	Filamentation	2.63**	8.16**
*HSP31*	Stress resistance	2.92**	10.18*
*GPX2*	Stress resistance	2.17**	1.28

*P < 0.05,

**p < 0.01

Gene expression was normalized using the average of 4 housekeeping genes (*ACT1*, *PMA1*, *RIP*, *RPP2B*)

#### Biofilm and filamentation-specific gene expression

Filamentation is an intrinsic part of biofilm formation in *C. albicans*, and both processes share key transcriptional regulators [53]–[58]. Genes involved in biofilm formation/filamentation that were differentially expressed in spaceflight conditions include *TUP1* (†), *ALS1*, *CPH1* (†), *AOX2* (†), and ORF19.4653. The latter gene was upregulated 7.5-fold in spaceflight, and is one of the ten most induced genes in the microarray. Interestingly, expression of the yeast-specific gene Yeast Wall Protein 1 (*YWP1*) was significantly induced in spaceflight samples, which promotes the non-filamentous phenotype of *C. albicans* under conventional culture conditions [59]. Additional genes involved in *C. albicans* biofilm formation (as determined by the GO Slim Mapper) that were differentially regulated by spaceflight include *BRG1*, *MCR1*, *RHR2*, and *SHA3* [50]. Additional spaceflight-induced genes involved in hyphal growth (as determined by the GO Slim Mapper) include *FGR16*, *ARC40*, *RFX2*, *SHA3*, *SPT5*, *STE13*, *TCA5*, *VID27*, and orf19.1617 [50].

Next, we analyzed the expression of genes involved in the production of biofilm-associated extracellular matrix proteins. The gene encoding the glucanosyltransferase Phr1 (†), involved in glucan modification [60] was significantly upregulated in spaceflight conditions. As indicated by light microscopy and flow cytometry (see below), spaceflight-grown *C. albicans* showed enhanced self-aggregation as compared to ground controls. Since the observed cell aggregation in spaceflight-grown *C. albicans* structurally resembles the well-characterized flocculation phenotype of *S. cerevisiae*, we investigated whether genes involved in flocculation were differentially expressed. The cell surface glycoprotein Als1, which is both involved in self-aggregation of *C. albicans* and has both structural and functional similarity to the main flocculation protein Flo11 in *S. cerevisiae* [61], [62], was induced in spaceflight conditions. In addition, a gene encoding a protein similar to cell surface flocculin (*HYR10*) (†) was induced in spaceflight cultures. Genes involved in the three main flocculation regulatory pathways (based on the well-characterized *S. cerevisiae*) were found to be differentially regulated in spaceflight-cultured *C. albicans*. For MAPK-dependent filamentous growth, these genes were *TPK1* (†) (Ras-cAMP pathway), the ammonium permease Mep2, and the transcriptional regulator *CPH1* (homolog of Ste12 in *S. cerevisiae*) (†). For the glucose repression pathway, these genes were *HXT3* (†), *HXT5*, *HGT1* and *HGT2* (all hexose transporters).

#### Stress and drug resistance

A significant portion of genes within the stress/drug response categories were related to oxidative stress resistance. The gene encoding the oxidative stress response transcriptional regulator, Cap1, was significantly induced in response to spaceflight culture. Interestingly, more than 30% of the previously reported Cap1 regulon [63] was affected by culture of *C. albicans* under spaceflight conditions in this study. This includes genes under positive Cap1 control: *TRX1*, *SOD1*, *PDR16* (†), *IFR1*, *ARR3*, orf19.7042, *ARO9* (†), *YIM1*, *RIB1* (†), orf19.1162 (†), *ADH6*, *ESBP6*, *HGT2*, orf19.6464 (†); and negative Cap1 control: *MNN13*, *VMA10* (†), *CHA2* (†). Among these 17 genes, 13 were expressed in the expected direction (i.e., *TRX1*, *IFR1*, *ARR3*, orf19.7042, *ARO9*, *YIM1*, *RIB1*, orf19.1162, *ESPB6*, *HGT2*, *MNN13*, *VMA10*, and *CHA2*). Additional spaceflight-induced genes identified in this study that have been reported to play a role in the oxidative stress response of *C. albicans* via Cap1 are *GZF3* and orf19.2498 [50]. Other genes involved in the oxidative stress resistance of *C. albicans* that were induced in spaceflight include *GPX1* and *GPX2*, which encode glutathione peroxidases; and *SOD3*, which encodes a superoxide dismutase.

Furthermore, genes encoding the heat shock proteins Hsp10, Hsp30, Hsp31, Hsp60, Hsp78, Mdj1, Ssc1, orf19.9899 (putative heat shock protein), and Sti1 were significantly upregulated in spaceflight-cultured *C. albicans* cultures.

In addition, spaceflight cultures of *C. albicans* showed significant upregulation of genes encoding ABC transporters and major facilitators, which are two main classes of drug transporters in *C. albicans*. These include *CDR1* (†), *CDR4*, *CDR12*, *HOL4*, *HOL2* (†), ORF19.4779, *YOR1* (†), and orf19.10632 (possible ABC transporter). Spaceflight cultures also showed significant downregulation of the ergosterol-encoding genes *ERG6* and *ERG25* (reviewed in [64]), of which *ERG6* has been shown previously to be important for amphotericin B resistance (a polyene) in *C. glabatra* [65], [66].

#### Bud site selection and cytoskeleton

Since we observed a higher abundance of random budding in *C. albicans* cultures exposed to spaceflight using SEM analysis (see below), we screened the microarray results for differential expression of genes involved in unipolar, axial, and random budding, as identified by Ni *et al*. for *S. cerevisiae* [67]. With the exception of the downregulation of *ALG5* and *BUD7*, which are involved in unipolar and axial budding respectively, a significant number of differentially expressed genes following spaceflight culture were involved in random budding. These differentially expressed genes were classified in the categories of vesicular transport (downregulation of *CLC1*, *VMA5* (†), *VPS34* (†), *VAC7* (†), *END3* (†), *LUV1* (†), *VPS45* (†), *SEC22*), actin cytoskeleton (downregulation of *SLA1* (†)), cell wall proteins (upregulation of *GAS1*), lipid metabolism (downregulation of *FEN1* (†)), protein modification (downregulation of *PMT2* (†), *OST3*; upregulation of *MAP1*), transcriptional proteins (upregulation of *CTK1* (†) and *TUP1* (†)), nuclear proteins (downregulation of *TRF4* (†); upregulation of *NPL3* (†), *SFP1* (†)), and other proteins (downregulation of *ATP14* (†) and *ILM1* (†)). Interestingly, induction of the gene encoding the daughter-cell specific transcription factor Ace2 [68] was observed for spaceflight samples of *C. albicans*. Accordingly, downregulation of the gene encoding the G1 cyclin Cln3, which is under the negative control of Ace2, was observed [69]. Given the essential role of the actin cytoskeleton in random budding and previous findings that microgravity profoundly affects the mammalian cytoskeleton [70], we screened our microarray data for additional genes involved in the actin cytoskeletal organization [50]. We discovered significant downregulation of several key genes involved in actin polymerization and organization, including *PFY1* (†), *SLY1* (†), *FAC1* (†), *ACF2*, *AIP1* (†), AND *SDA1* (†). Accordingly, differences in cell size and shape were observed when *C. albicans* was grown in spaceflight and ground conditions (see below).

#### RNA-binding proteins

A high percentage of differentially expressed genes in the GO Slim Mapper analysis were assigned to categories related to metabolism (**Table 3**). We were particularly interested in genes assigned to ‘RNA metabolic processes*’* (GO:0016070) based on the identification of the RNA binding protein Hfq as a global regulator of microgravity and/or microgravity-analogue culture induced responses in *S.* Typhimurium, *P. aeruginosa*, and *S. aureus* [11], [20], [21].

The eukaryotic LSm proteins share structural and functional similarities with their prokaryotic counterpart, Hfq [71], [72]. The gene encoding LSm2 (†) was the only LSm family member observed to be differentially expressed in response to spaceflight culture under the conditions of this study. We considered the possibility that other RNA-binding proteins may be differentially expressed upon exposure to microgravity; therefore, the GO Slim Mapper ‘function’ category of RNA-binding proteins was investigated, which allowed us to identify 12 additional genes involved in RNA binding whose expression was significantly altered in response to microgravity culture, i.e., *PRP39*, *SPT5*, *STI1*, *TCA5*, *YTH1*, orf19.2610, orf19.265, orf19.3114, orf19.3547, orf19.4479, and orf19.6008. Interestingly, the genes encoding Yth1, Prp39, Spt5, Sti1, and Tca5 have been associated with hyphal formation [50].

### Morphological analyses

**Figure 3:**
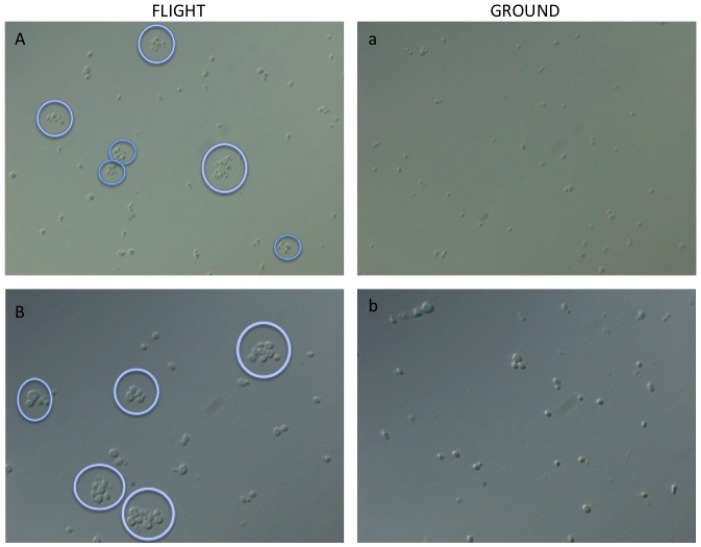
Light microscopic analyses of fixed *C. albicans* cultured in spaceflight (A, B) and ground control (a, b) conditions. Panels A and B: Differential interface contrast (DIC) images at 400× magnification. Panels a, b: DIC images are 630× magnification. Purple circles indicate cell clumps of 4 or more cells.

**Figure 4:**
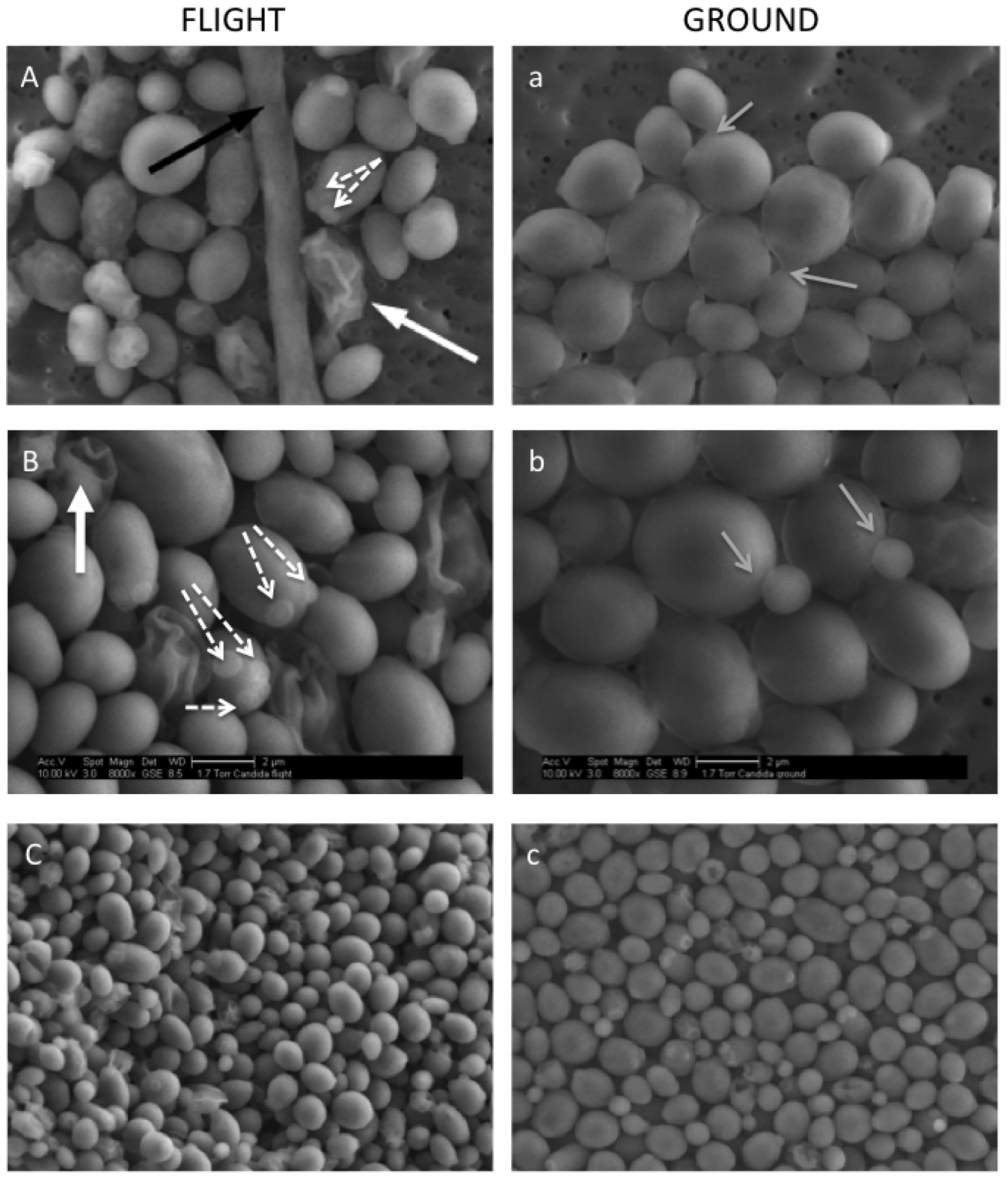
Scanning electron microscopy analysis of *C. albicans* cultured in spaceflight and ground control conditions. Cell clusters of spaceflight (A, B) and ground control (a, b) conditions are shown. Black arrow points to filament, white arrows indicate aberrant cell shapes, grey arrows indicate normal bipolar budding, and white dotted arrows indicate random budding scars. Magnification  = 5,000× for A and a, and 8,000× for B and b. C and c show images of spaceflight and ground control cells respectively at lower magnification (2,500×) to demonstrate the difference in space occupancy between the test conditions (3D architecture for spaceflight compared to flat structure for ground cultures).

Light microscopic analysis revealed enhanced cellular aggregation in flight samples as compared to synchronous ground controls (**Figure 3**). While both flight and ground cultures showed cell clumping and occasional filamentation, cell cluster formation was more pronounced in flight samples of *C. albicans*. Based on microscopic imaging, spaceflight samples contained more cell clusters and their average size was larger compared to synchronous ground controls (1.7-fold, 10±3 cells per cluster for flight samples versus 6±1 cells per cluster for ground samples). In both test conditions, some cell clusters contained one filament (**Figure 4A**, black arrow). **Figure 4** shows 2500×, 5000× and 8000× SEM images of cell clusters from flight (A, B, C) and ground samples (a, b, c) respectively.

**Figure 5:**
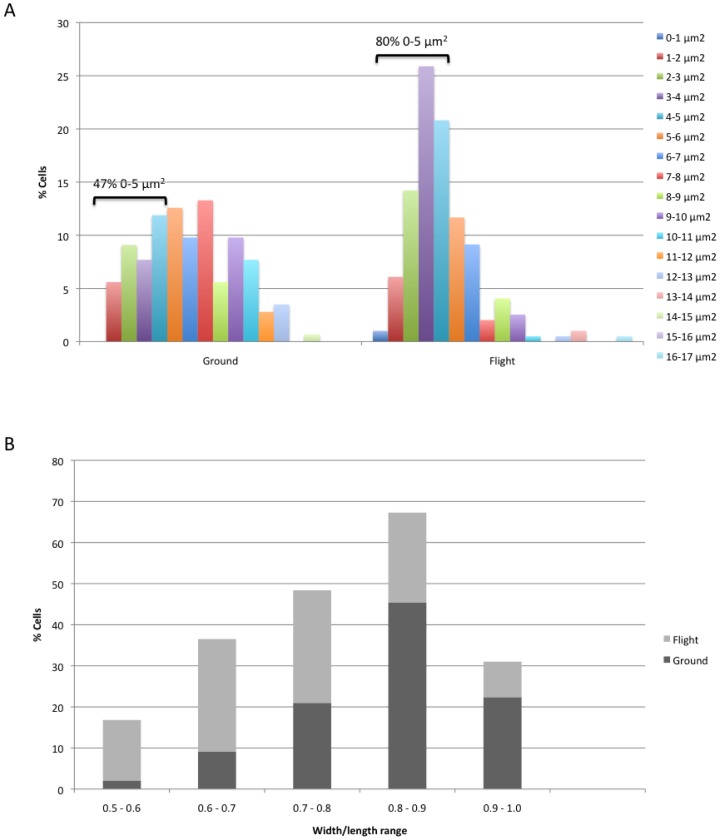
Measurement of cell size and shape of *C. albicans* spaceflight and ground control cultures. (A) Surface area of spaceflight and ground cells, organized as percentage of cells per size range (1 µm increments). The percentages for ground and flight cultured *C. albicans* with a surface area between 0 and 5 µm are indicated. (B) Width-to-length ratio of spaceflight and ground cells, organized as percentage of cells per width-to-length range (0.1 increments). Results were obtained based on surface area and width-to-length determination of 143 ground control cells and 197 spaceflight-cultured cells.

*C. albicans* ground samples exhibited a higher number of cells with a bipolar budding pattern (reflected by ongoing budding and budding scars), while more cells with multiple, randomly distributed budding scars were observed for spaceflight cultures (**Figure 4A** **and a**, white dotted arrows). Accordingly, genes involved in random budding of *C. albicans* were significantly affected by spaceflight culture. Since the multiple budding phenotype could indicate the generation of more daughter cells that are typically smaller, the cell surface area, width and length were determined for *C. albicans* cells grown in ground or spaceflight conditions, respectively. The average surface area for ground samples (6.6±3.0 µm^2^) was significantly higher than for flight samples (4.6±2.4 µm^2^) (1.4-fold, p<10^−9^). In addition, **Figure 5A** shows that a higher percentage of cells with a smaller surface area was observed for spaceflight cultures. For example, 80% of the spaceflight cells versus only 47% of ground cells had a surface area smaller than 5 µm^2^ (**Figure 5A**). To assess cell shape, we determined the width-to-length ratio. Ground control cells had a higher percentage of cells with a ratio above 0.8 (67.8% for ground versus 30.5% for spaceflight), indicating that more *C. albicans* cells grown in control conditions had a rounder morphology (**Figure 5B**). It is important to note that ground control cells appeared more flat, compared to spaceflight cells, which showed a 3D organization (**Figure 4C** **versus 4c**). This could potentially explain, at least in part, a larger surface area for ground control cultures. Also, the increased presence of aberrant yeast forms was observed in spaceflight samples (**Figure 4A** **and a**, white arrow). The aberrant yeast forms in panels A and a are reminiscent of dying cells. However, post-flight viable cell counts indicated no differences between cultures exposed to microgravity and synchronous ground controls (i.e., 4.78×10^7^ CFU/mL for flight samples and 5.94×10^7^ CFU/mL for ground samples).

**Figure 6:**
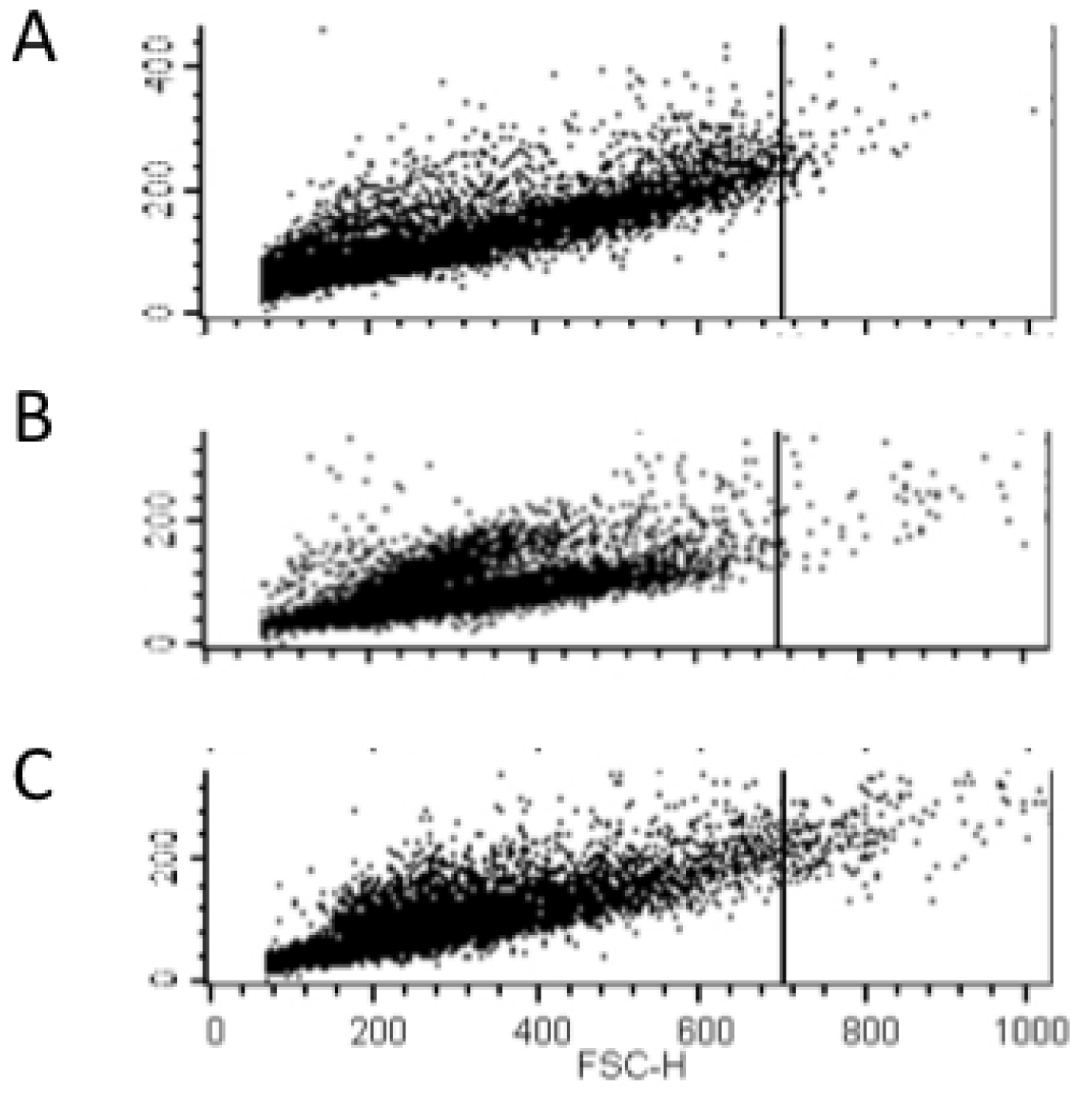
Flow cytometry analysis of *C. albicans* flight samples and ground controls. Panel A represents a dot plot of *C. albicans* yeast cells grown at 30°C (to set the threshold for non-flocculated organisms). Panels B and C illustrate dot plots of ground and flight samples respectively. The Y-axis represents side-scatter and the X-axis forward scatter (FSC). Events with FSC values below the established threshold were considered single or budding yeast, whereas events above the established threshold were considered cell clusters.

Flow cytometry analysis demonstrated a 2.8-fold increase (p<0.025) in forward scatter signal for spaceflight-grown *C. albicans* (**Figure 6**), which is reflective of the observed increases in cell aggregation in spaceflight samples.

### Virulence

Due to limited sample availability, a focused study to determine the effect of spaceflight culture on *C. albicans* virulence was performed by infecting mice via the i.p. route with a single infection dose grown under spaceflight/ground control conditions and monitoring the time to death. This targeted study indicated no differences between the virulence of spaceflight and ground cultures, as reflected in comparable mouse survival in both test conditions (**Figure S2**).

## Discussion

The presence of the opportunistic fungus *C. albicans* in the normal flora of astronauts could present an infectious disease risk during long-term missions. Indeed, microorganisms have been shown to enhance their virulence and/or display virulence-related phenotypes in response to culture in the low fluid-shear environment of both microgravity and microgravity-analogue culture systems [10]–[12], [20]–[22], [24], [34], [35], [73]–[79]. Moreover, as *C. albicans* causes a variety of mucosal and deep tissue infections in immunosuppressed patients [9], the decreased immune response of astronauts in-flight could further contribute to an increased susceptibility to microbial infections [1].

In addition to the application of spaceflight microbiology studies for infectious disease risk assessment in the astronaut population, these studies also entail applications to advance human health on Earth. Complementing conventional infectious disease research with spaceflight studies can serve to bridge gaps in our current understanding of host-pathogen interactions, given the unique ways in which both the host and pathogen respond to this extreme environment [1], [2], [24]. The low fluid-shear forces to which microorganisms are exposed during liquid culture in spaceflight and spaceflight analogues is relevant to environmental conditions encountered during their normal terrestrial lifecycles, including in the gastrointestinal, respiratory, and urogenital tracts of the host [3], [42]–[45]. Thus, studying the responses of microbial cells to the microgravity environment of spaceflight holds potential for the discovery of novel infectious disease mechanisms that cannot be observed using conventional culture conditions, where the force of gravity can mask key cellular responses.

This study demonstrated that spaceflight culturing induced a self-aggregative phenotype (resembling the flocculation phenotype of *S. cerevisiae*) in *C. albicans* and altered a plethora of genes involved in stress and drug resistance; which is important for the virulence of this organism. The high prevalence of differentially expressed genes involved in biofilm formation and filamentation of *C. albicans* in response to spaceflight culture suggests that the microscopically observed self-aggregative phenotype could be reflective of biofilms. Indeed, transcriptional regulation of biofilm formation and filamentation is intertwined in *C. albicans*, and an increased flocculation phenotype is believed to be the result of hyphae-specific gene expression [80]. *C. albicans* biofilm formation is divided into four distinct phases: (i) surface adhesion and colonization by yeast-form, spherical cells, (ii) microcolony formation on the attached surface by yeast-form cells, (iii) growth of pseudohyphae and hyphae in concert with synthesis of extracellular matrix, and (iv) dispersal of yeast-form cells to initiate biofilm formation off-site [53], . Microcolony formation on abiotic surfaces (structurally similar to flocculation) is estimated to take place 3–4 hours after initial adhesion, while formation of pseudohyphae and hyphae occurs at later time points (12–30 hours) [54]. We hypothesize that at the 25-hour time point of fixation in this study (for gene expression/microscopic analysis), *C. albicans* may have been in the process of transitioning to the hyphal biofilm stage, which was not yet translated at the phenotypic level. In support of this hypothesis is the previous finding that *C. albicans* grown in LSMMG conditions exerted increased biofilm formation and biofilm-associated filamentation after long-term culture in the RWV bioreactor (4–5 days) [35]. In microgravity-analogue conditions, biofilm formation was observed on the gas-permeable siliconized rubber membranes of RWV bioreactors, while in spaceflight samples, self-aggregation of microbial cells was observed. Interestingly, flocculation of *S. cerevisiae* has also been reported in LSMMG conditions, but detailed analysis of gene expression was not performed [32]. Furthermore, *P. aeruginosa* and *S. aureus* grown in LSMMG also displayed self-aggregative biofilm phenotypes [20], [73], and *S.* Typhimurium formed biofilms during spaceflight culture [11]. For *C. albicans*, key regulators of filamentation that were differentially regulated by long term culture in LSMMG (i.e., repression of *YWP1*, induction of *HWP1* and *BCR1*) were not differentially expressed in shorter term spaceflight-grown *C. albicans*; although the gene encoding the cell surface glycoprotein Als1 showed significant induction in both spaceflight and spaceflight-analogue cultures. Als1 is functionally and structurally similar to the major flocculation protein in *S. cerevisiae*, Flo11, and is an effector of filamentation, and a mediator of adherence and flocculation [62]. The transcriptional regulation of self-aggregation has extensively been studied in *S. cerevisiae* given the associated industrial applications of this phenotype. Three main pathways have been proposed to regulate flocculation (via Flo11) in *S. cerevisiae*: (i) Ras-cAMP, (ii) MAP kinase (MAPK)-dependent filamentous growth, and (iii) main glucose repression pathway [82]. In this regard, genes involved in the three main flocculation regulatory pathways were also found differentially regulated in spaceflight-cultured *C. albicans*. Therefore, Als1 could be a key mediator in the observed spaceflight-induced self-aggregative phenotype of *C. albicans*.

We also examined the expression of genes involved in the production of biofilm extracellular matrix proteins. While the complete composition and transcriptional regulation of the extracellular matrix of *C. albicans* biofilms remains to be unveiled, studies have shown the presence of carbohydrates, proteins and nucleic acid components [83]–[85]. A recent study identified three glucan modifying genes that play a role in glucan incorporation in the biofilm matrix [60], one of which, glucanosyltransferase (Phr1), was significantly upregulated in spaceflight conditions.

Another morphological change that was observed for spaceflight cultures of *C. albicans* was the presence of an increased number of cells with random budding scars as compared to more cells with a bipolar budding pattern for synchronous ground controls. This phenotype was also observed for *S. cerevisiae* exposed to spaceflight culture conditions [28]–[30]. Polarized cell division is essential for the development of eukaryotes and prokaryotes, and typically takes place at the distal cell poles (180° from the birth site), termed bipolar budding, or at the proximal cell poles (adjacent to the preceding site of cytokinesis), termed axial budding [86], [87]. Bipolar budding is believed to maximize nutrient exposure of the growing yeast cells [86], while axial budding facilitates mating and diploid formation [88]. Specific mutations and environmental conditions cause random budding which is associated with loss of cell polarity, as reflected in a round cell morphology and cell separation deficiency, associated with production of cell clumps [87], [89]. As described above, enhanced cell clumping was observed for spaceflight cultures of *C. albicans*. In agreement with the random budding phenotype of *C. albicans* in spaceflight cultures, multiple genes involved in random budding of yeast were significantly affected. Interestingly, the enhanced presence of multiple budding scars could indicate the generation of more daughter cells in spaceflight conditions, which is supported by the smaller cell size of spaceflight-cultured *C. albicans*, and at the transcriptional level, by the induction of the daughter-cell specific transcription factor *ACE2* and downregulation of the G1 cyclin *CLN3* in spaceflight-cultured *C. albicans* (see above) [68]. In yeast, asymmetric cell division results in the generation of smaller daughter cells as compared to the mother cell [90]. Since the regulation of the G1 cycle is, in part, dependent on cell size; daughter cells require additional growth before the Start transition in G1. This process is orchestrated by a cell size-sensing module, in which Cln3 is the main regulator [91]. The daughter-cell specific transcription factor, Ace2, has a direct negative regulatory effect on the expression of *CLN3*, which plays a role in delaying the G1 phase in daughter cells [69]. The enhanced presence of daughter cells could also indicate differential growth rate of *C. albicans* in spaceflight conditions. While at the time point of analysis, no differences in viable cell counts were recorded, more detailed monitoring of growth profiles are needed to determine if *C. albicans* altered its generation time in flight. It was hypothesized by Walther *et al*. that the random budding pattern in spaceflight cultures of *S. cerevisiae* could be explained by microgravity-induced changes in the cytoskeleton, which has been reported for a variety of mammalian cells (reviewed in [70]). Indeed, the actin cytoskeleton is essential for bud site selection, and mutants in actin organization exert a random budding phenotype [67]. In accordance with Walther and colleagues, we found that *C. albicans* exposed to spaceflight culture conditions downregulated several key genes involved in the actin organization and polymerization.

Several mechanisms of drug resistance have been described for *C. albicans* yeast cells, including differential expression of drug targets, efflux pump-mediated drug transport, and utilization of compensatory and catabolic pathways [64], [95]. Biofilm formation confers additional resistance in *C. albicans* through increased cell density, production of extracellular matrix proteins, and the presence of persisters [64], [96]. In this study, genes encoding ABC transporters and multidrug efflux proteins (major facilitator family) were induced in spaceflight-cultured *C. albicans* (such as *CDR1*, *CDR4, CDR12*), which are involved in resistance to different classes of antifungals including polyenes (e.g. amphotericin B) and azoles. Also, spaceflight cultures of *C. albicans* showed downregulation of genes encoding ergosterol (*ERG6*, *ERG25*), which is a major drug target for this organism. Ergosterol is uniquely present in the membranes of yeast and fungal cells, and polyenes specifically target ergosterol in the fungal membrane, which creates pores and results in cell death [95]. Downregulation of ergosterol levels in the cell membrane of sessile or biofilm-forming *C. albicans* contributes to the resistance of this organism to both polyene and azole antifungal agents. Interestingly, enhanced resistance of LSMMG-cultured *C. albicans* to amphotericin B was previously observed, which increased with the time of incubation under these microgravity-analogue conditions [35]. In addition, *S.* Typhimurium showed induction of outer membrane porins, ABC transporters, and other genes involved in antibiotic resistance in response to culture in spaceflight conditions [11]. Whether the observed differences in gene expression translate to a phenotype of *C. albicans* that is more resistant to antifungal drug agents remains to be determined.

We observed that a significant number of genes differentially regulated in response to spaceflight culture were involved in the oxidative stress resistance of *C. albicans*. Cap1 presumably played a role in the oxidative stress-associated gene expression since it has been shown to be involved in the oxidative stress response of *C. albicans* [63], and more than 30% of the Cap1 regulon was affected by spaceflight. It would seem unlikely that increased gene expression related to oxidative stress resistance is due to the presence of increased oxygen levels since previously reported gene expression profiles of bacterial FPA cultures exposed to spaceflight indicated responses to microaerophilic/anaerobic conditions, presumably due to low fluid-shear levels and/or limited mixing in microgravity [10], [11], [79]. In correspondence with our data, the spaceflight-induced proteome of *S. cerevisiae* comprised multiple proteins involved in oxidative stress [30]. Moreover, a recent study demonstrated that growth of *S. cerevisiae* in spaceflight in hyperoxic conditions resulted in extracellular release of glutathione [29]. The observed increase in glutathione release was suggested to have occurred through activation of ion channels in response to cytoskeletal rearrangements in microgravity culture conditions [29]. Spaceflight has been shown to modulate oxidative functions in other eukaryotic cell types, animal models, and astronauts [29], [97]–[102]. Collectively, our data indicate a potentially increased resistance of spaceflight-cultured *C. albicans* to antimicrobial agents and environmental stressors as compared to ground controls, which would need to be confirmed at the phenotypic level during future studies.

Despite the induction of a virulence-related phenotype of *C. albicans* in spaceflight conditions, we did not observe significant differences in virulence, as determined using an i.p. mouse model of infection. This observation could potentially be explained by the route of infection, the use of only a single lethal dose of *C. albicans* for the inoculation, and the short-term exposure to spaceflight. Indeed, i.p. infection is not a standard infection method for *C. albicans*, and was chosen given the unique time constraints associated with the spaceflight experiment. Alternatively, it is possible that spaceflight culture does not impact the virulence of *C. albicans*. Additional studies are needed to conclusively determine if spaceflight alters *C. albicans* virulence.

Since the RNA-binding protein, Hfq, was previously identified as a major regulator of the microgravity and/or microgravity-analogue response of *S.* Typhimurium, *P. aeruginosa* and *S. aureus* [21], we investigated the influence of spaceflight on expression of the LSm family of RNA-binding proteins in *C. albicans*, which are evolutionarily conserved eukaryotic homologues of Hfq [103]. The gene encoding the LSm2 protein was the only LSm family member that was significantly affected by spaceflight culture of *C. albicans* under the conditions of this study. LSm2 is part of (i) the cytoplasmic LSm1-7 complex, which is important for mRNA decapping and decay, and (ii) the nuclear LSm2-8 complex, which is important for pre-mRNA and pre-rRNA processing [104]–[107]. In response to stress, there is a rapid shift of LSm proteins from the nucleus to the cytoplasm where the LSm1-7 complex concentrates within granular foci called processing bodies (P-bodies) [104]–[108]. To our knowledge, the role of LSm2 in the transcriptional regulation, virulence and behavior of *C. albicans* is unknown. Whether LSm2 regulation is involved in the spaceflight response of *C. albicans*, supporting a conserved transcriptional regulation between prokaryotes and eukaryotes, needs to be assessed in follow-up studies.

In summary, this study is the first to demonstrate that spaceflight culture conditions globally alter the gene expression profile of a eukaryotic pathogen and could potentially induce a virulence-related phenotype, and represents an initial step towards the infectious disease risk assessment of *C. albicans* during spaceflight missions. The effect of longer-term microgravity cultivation on the biofilm formation, filamentation and virulence phenotype of *C. albicans*, together with investigation of the potential spaceflight-activated transcriptional regulator Cap1 identified in this study is of interest for future research. Moreover, this study further reinforces the role that physical forces in the human body, such as low fluid-shear, could play in the infection process; insights that hold promise to fundamentally advance our understanding of infectious disease on Earth.

## Supporting Information

Figure S1 **Schematic of fluid processing apparatus (FPA).** FPAs were used to initiate growth of *C. albicans* in spaceflight and ground control conditions (*activation*) and to fix *C. albicans* following growth in spaceflight and ground control culture conditions (*termination*). Panel A: The pre-flight assembly of the FPA with *C. albicans* in stationary phase. Panel B: The post-flight FPA in which *C. albicans* has been grown for 25 hours in space and on the ground and then fixed. Black boxes represent rubber stoppers, and grey boxes represent gas exchange membranes.
(JPG)

Figure S2 **Percent survival of mice following i.p. infection with** ***C. albicans*** **cultured in spaceflight and ground control conditions.**
(PDF)

Table S1 **Surface area, width and length measurements of** ***C. albicans*** **grown in spaceflight and ground control conditions.**
(XLS)

Table S2 **Complete microarray gene list.**
(XLSX)
