# Supplementary figures and images for: Spaceflight Enhances Cell Aggregation and Random Budding in Candida albicans
Source: PLoS One. 2013 Dec 4;8(12):e80677. doi: 10.1371/journal.pone.0080677 (PMC3851762; doi:10.1371/journal.pone.0080677)

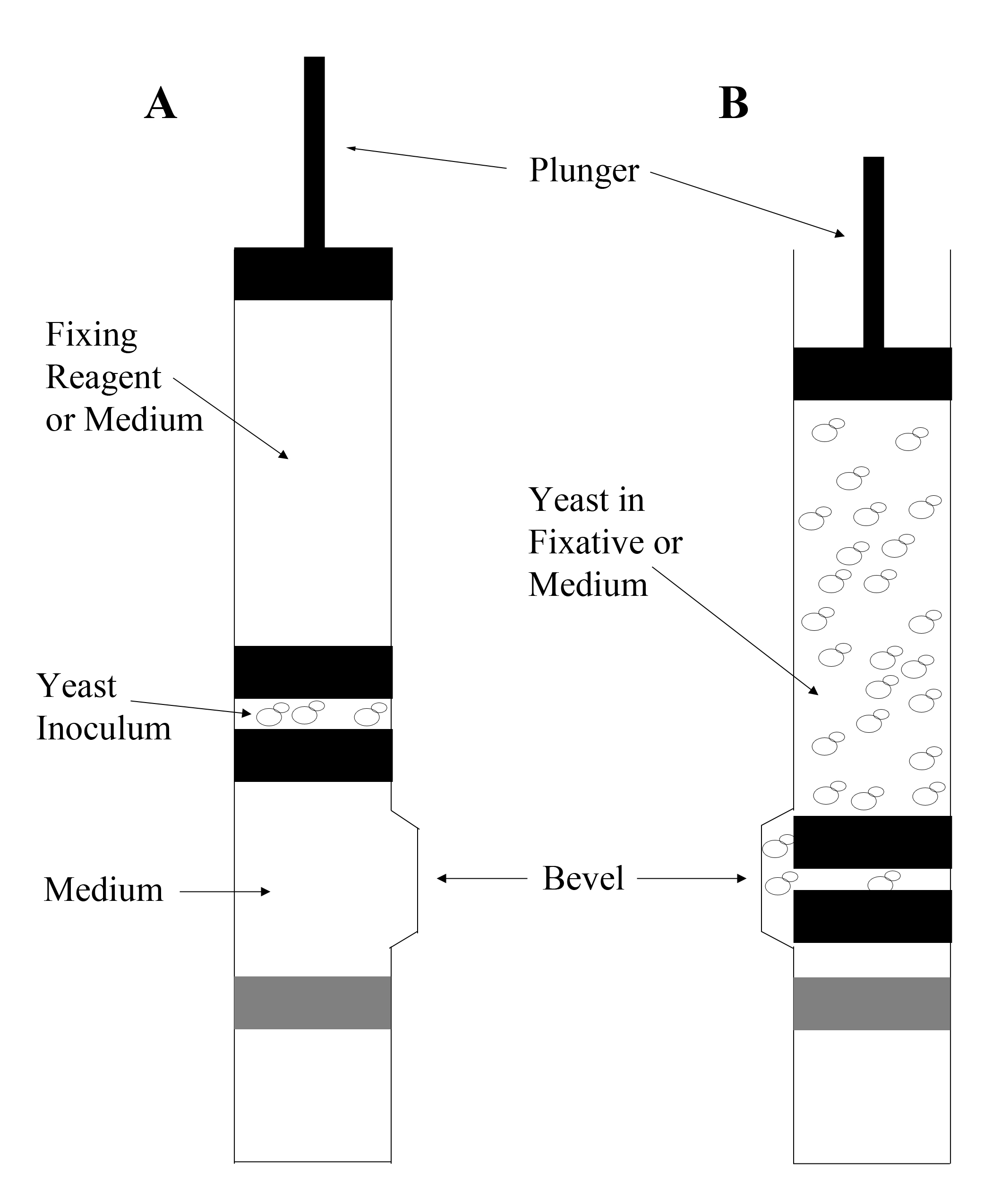

Supplement: Figure S1 — Schematic of fluid processing apparatus (FPA). FPAs were used to initiate growth of C. albicans in spaceflight and ground control conditions (activation) and to fix C. albicans following growth in spaceflight and ground control culture conditions (termination). Panel A: The pre-flight assembly of the FPA with C. albicans in stationary phase. Panel B: The post-flight FPA in which C. albicans has been grown for 25 hours in space and on the ground and then fixed. Black boxes represent rubber stoppers, and grey boxes represent gas exchange membranes. (JPG) [file pone.0080677.s001.jpg]

Percent survival

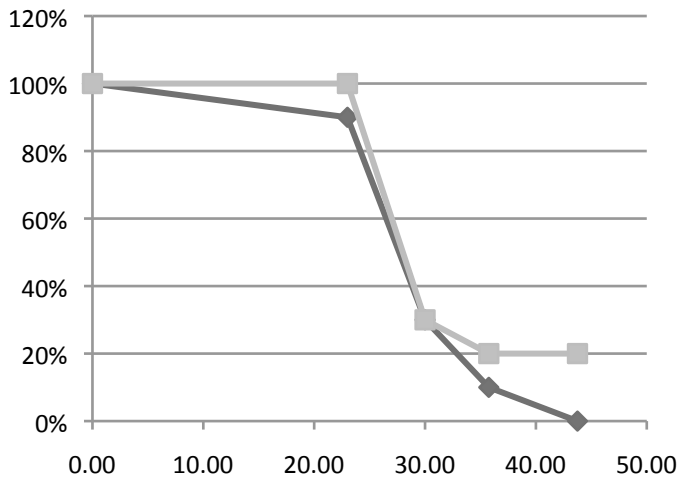

Hours post-infection

Ground  
Flight

Supplement: Figure S2 — Percent survival of mice following i.p. infection with C. albicans cultured in spaceflight and ground control conditions. (PDF) [file pone.0080677.s002.pdf]
